# Research progress on the applications of paper chips

**DOI:** 10.1039/d0ra10470a

**Published:** 2021-02-26

**Authors:** Xin Tong, Lu Ga, Ruiguo Zhao, Jun Ai

**Affiliations:** College of Chemistry and Enviromental Science, Inner Mongolia Key Laboratory of Green Catalysis, Inner Mongolia Normal University 81 zhaowudalu Hohhot 010022 China imacaj01@163.com; College of Pharmacy, Inner Mongolia Medical University, Jinchuankaifaqu Hohhot 010110 China; College of Chemistry and Chemical Engineering of Inner Mongolia University Hohhot 010020 China

## Abstract

Due to the modern pursuit of the quality of life, science and technology have rapidly developed, resulting in higher requirements for various detection methods based on analytical technology. Herein, the development, fabrication, detection and application of paper-based microfluidic chips (μPAD) are summarized. We aim to provide a comprehensive understanding of paper chips, and then discuss challenges and future prospects in this field.

## Abstract 2

Due to the modern pursuit of the quality of life, science and technology have rapidly developed, resulting in higher requirements for various detection methods based on analytical technology.

## Graphical abstract

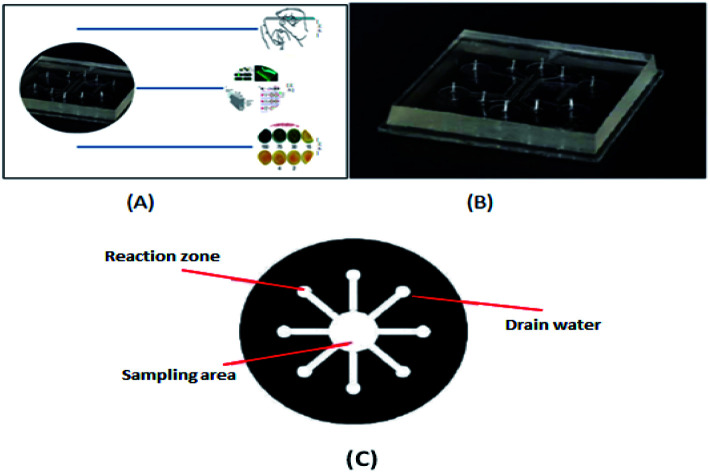

## 1 Introduction

With the continuous development of modern science and technology, analytical technology is becoming increasingly ideal, starting from the perspective of daily life, which has greatly changed the outlook of society and scientific research. Paper chips are becoming increasingly popular as analysis and detection devices, mainly because of their advantages such as low cost, simple operation, strong portability and mass production. In recent years, great progress has been made in the research on paper functionalization, paper chip fabrication methods and analysis methods. Furthermore, many new reports on paper research are emerging. This review focuses on the research progress of paper-based microfluidic chips, providing comprehensive and important insights into the manufacturing materials, fabrication methods, detection methods and applications of paper chips.

## 2 Microfluidic chip technology

### 2.1 Introduction to microfluidic chip technology

**Fig. 1:**
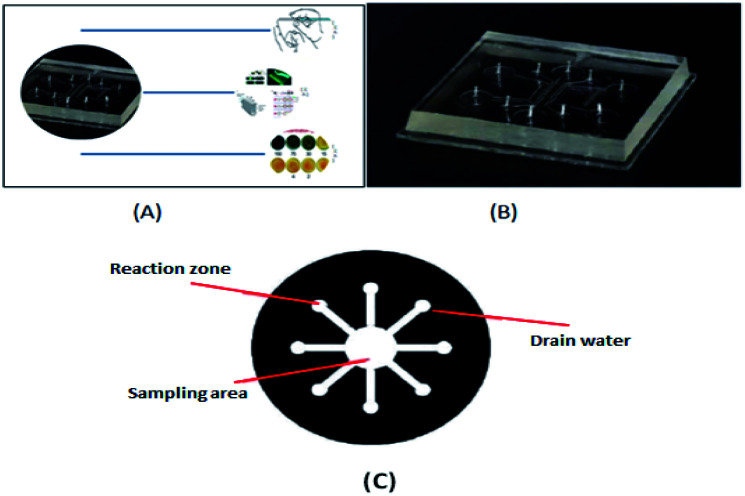
(A) Schematic diagram of microfluidic chip application. (B) Schematic diagram of a microfluidic chip. (C) Micro-complete analysis equipment.

The prototype of microfluidic chip technology is micro-electro mechanical system (MEMS) technology.^1–7^ Originating from late 1970s, Terry *et al.* realized the construction of micro-scale gas chromatography on silicon wafers and demonstrated the effect of this device on the composition analysis of mixed gases. As shown in Fig. 1(A), the current research and application of microfluidic chip technology^8^ involve many fields such as life sciences, chemistry, materials science, biosynthesis, biochemical diagnosis and analysis, and drug screening. Recently, in the study by Mohammad Baharloo *et al.*,^9^ to effectively improve the network-on-chip (NoC) performance, they used the applied power in a multiple network-on-chip (multi-NoC) to replace the traditional single network-on-chip (single-NoC), and used the ChangeSUB framework to change the hardware circuit of the subnet, thus avoiding the network performance decline caused by closed routers. Zheng Xiao's group^10^ achieved the aim of scheduling threads fairly by breaking threads into multiple scheduling batches to degrade the balance of the system. The linear relationship between the L2 cache miss ratio of threads and the total system performance degradation was established to predict the degradation in the system performance under the same batch of scheduled threads, and the cooperative scheduling of threads on the chemical mechanical polishing architecture was considered according to the game theory. Cleverly, they selected the right set of threads to run on the processor. This thread scheduling can minimize conflicts and interference between threads, minimize contention for shared caches, and maximize utilization and system performance. The common driving modes used in microfluidic biochemical analysis systems are centrifugal drive, electric drive, pressure drive, and capillary drive, in addition to magnetic drive, hot capillary migration, acoustic drive and other driving modes. In 1990, the miniaturized total chemical analysis system (μTAS) was first proposed by A.MANZ and coworkers in Sweden. This system is also called lab-on-a-chip (LOC).^11,12^ Microfluidic chip technology is a type of liquid sample or detection reagent on the micrometer scale (10–8–10–9 L) controlled by a micrometer-level or submicron-level channel to form a microflow path under the action of a pressure pump or electric field. As shown in Fig. 1(B and C), one or more continuous reactions are carried out on the chip to achieve high-throughput rapid analysis. Microfluidic devices not only can reduce the amount of samples and reagents, but also reduce the complexity of the operation, and greatly shorten the detection time under the condition of ensuring specificity and sensitivity, which is the reason why they are attracting increasing attention.

### 2.2 Characteristics of microfluidic technology

① Micro-multifunction. The detection system is very small, with the entire chip covering only a few square centimeters, but it has many functions that cannot be achieved using a macro system at the micron or even the picometers size. ② Low cost and low consumption. Micron-level channels and miniaturization analysis methods reduce reagent consumption to the microliter level and even upgraded for use on human skin, which not only reduces the consumption of reagent and cost, but also reduces waste liquid pollution. ③ High detection speed, high sensitivity, high flux and high efficiency.^13^ In the scale of microliter and even upgrade to human skin, the short distance and high heat transfer rate of the solution are conducive to improving the reaction efficiency, shortening the reaction time and analysis time, and realizing multiple operations, which can be completed in a very short time. ④ It has a high degree of automation and integration. It has a variety of functional units conducive to the integration and assembly of miniaturization and can realize various multi-channel and parallel unit detection, and thus microfluidic chips can analyze and process a large number of samples in parallel. ⑤ Because of its small size, it can be made into a portable instrument for temporary field analysis.

## 3 Paper-based materials

Paper materials^14,15^ have the unique properties of separating and supporting in microfluidic devices. Paper-on-paper analysis with strips as an example is applied to a large number of rapid and simple diagnostic tests without the assistance of external equipment. Thus, in remote areas, people can be quickly diagnosed and the results can save many lives. Harvard University Professor George Whitesides revolutionized microfluidics technology, where combined with the low cost, easy operation, and convenient transportation of paper, multi-functional one-time test kits were produced, which require only a small amount of blood or urine to diagnose chronic diseases and infections.

## 4 Paper chips

Paper chips can be classified into three types, including test paper analysis, transverse flow mode paper chips (LAFs) and paper microfluidic chips (PADs).

### 4.1 Test paper analysis

Test paper analysis is one of the simplest applications of paper chip analysis devices. The principle is to pre-imprint the reagent that responds to the test object on the surface of paper. Dipstick analysis was first developed by the Parisian chemist Jules in 1850 for the detection of urine, and later marketed by the British physiologist George Oliver in 1883. In 1932, Martin and Singer used paper as a reaction carrier to invent the first paper chromatography with paper as a sensor and won the Nobel Prize in Chemistry. The earliest use of paper-based materials was the discovery in the 17th century by an English chemist named Robert Boyle, where a purple extract from litmus became red when it was acidic and blue when it was alkaline, who used this characteristic to make acid–base test paper, namely litmus paper. Then various pH test papers represented by litmus paper were successively created and produced for qualitative analysis and applied in the fields of medicine, industry and biochemistry. In the 1920s, pH dipstick was commercialized and gradually developed into a simple three-dimensional qualitative tool commonly used in the chemical laboratory to determine the acidity and alkalinity of reagents during experiments.

### 4.2 Transverse flow mode paper chips (LAFs)

In the transverse flow mode,^16–18^ all the reagents are stored in a certain area first, and as the sample size increases, they reach the reaction area through capillary action. The advantage of this model is that the sample passes through different reaction areas with different reagents, and these different reaction areas have different functions. A specific transverse flow measurement is generally made up of four distinct components, namely a sample gasket, a coupling gasket, a test pad, and an absorbent pad. The function of the sample gasket is to filter impurities by cellulose as a filter when the sample reaches the reaction area. The function of the bonding pad is to store the reagent for the reaction, which it is made of glass fiber. The coupling gasket has the function of fixing and capturing signals, which is composed of nitro fiber membrane. The function of the absorbent pad is to allow the liquid to pass through the membrane under capillary action to reach the reaction area, thus improving the sensitivity. It is composed of cellulose filter. The most commonly used detection method is colorimetric detection, and the most common example is the pH test paper. However, the disadvantage of this method is that it cannot simultaneously carry out multi-sample analysis or quantitative analysis and it is limited to routine detection, not complex experiments.

### 4.3 Microfluidic paper chips (μPADs)

Microfluidic paper chips^19^ overcome the difficulty of simultaneous qualitative analysis and quantitative analysis of multiple samples in transverse flow mode paper chips. Also, this method does not need any pump or external energy sources, is in the closed water channel (hydrophobic) paper to create a hydrophobic (pro) water channel, and relies on the capillary action in the paper itself to make the liquid flow channel. Also, it can detect the samples with a very low concentration and be used for the testing and quantitative analysis of many samples simultaneously. In 2009, the paper chip was rated by Technology Review as one of the top 10 emerging technologies (Table 1).

**Table 1:** Development of paper-based materials

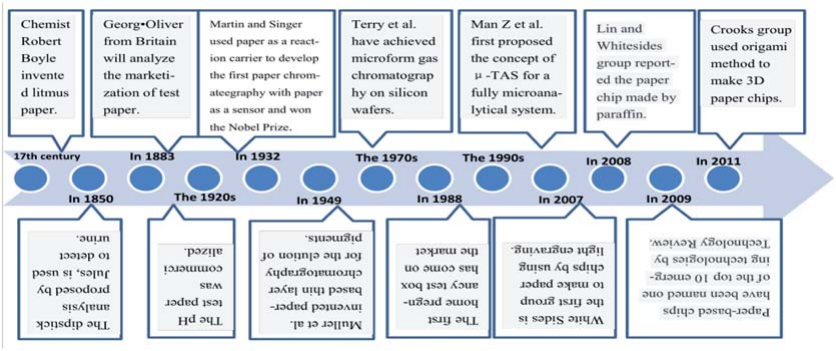

## 5 Microfluidic paper chip technology

### 5.1 Overview of microfluidic paper chips

Paper-based microfluidic chips^20^ use paper-based substrate materials to design hydrophilic or hydrophobic channels through a series of fine processing technologies to construct “lab on a chip”, namely “microfluidic paper-based analytical devices (μPADs)”. Microfluidic paper chips and their concept were first proposed by Whitesides of Harvard University and his colleagues in 2007.^21^ The paper microfluidic chip can overcome the characteristics of simultaneous qualitative and quantitative analysis of multi-component objects that traditional test paper cannot achieve. As a new type of personalized diagnostic device, paper-based microfluidic chips^22^ meet the requirements of mass production for point of care testing (POCT). Thus, researchers have paid great attention to them and there is great demand for this type of portable sensor, which has been gradually applied in life sciences, chemistry, drug screening and other research fields. Given that the working principle of paper chips is that paper material is divided into hydrophilic and hydrophobic zones in a specific way and basic operating units such as sampling, separation, reaction and detection are formed, it is of great importance to establish hydrophilic channels and hydrophobic channels to prevent the random flow of samples. Accordingly, due to the growing number of studies on the performance of the surface of paper, such as capillary effect factors, in-depth research is expected to reveal different types of factors and their role and influence of microfluidic technology. For example, the internal factors to improve the sensitivity and accuracy of paper-based microfluidic chips, especially for clinical medical examination in some remote areas is of great significance.

### 5.2 Mechanism of paper as a substrate

The main reason why paper acts as a microfluidic substrate is that it has its own capillary structure, and due to the characteristics of cellulose,^23^ paper has a unique property in detection devices, where it can play a separation and support role.^24,25^ Thus, paper-based materials are the substrate of choice for microfluidic chips.

### 5.3 Choice of paper

The choice of the paper material is critical, and multiple factors need to be considered^26,27^ such as it should have enough tolerance, good heat dissipation and electrical insulation. Also, no significant deformation and disintegration should be observed after immersion in the liquid phase. Paper has appropriate hydrophilicity and good modifiability, which facilitate the adsorption and fixation of the substances to be measured, thus forming a clear detection area and providing more sites for the fixation of large particle biomolecules, while avoiding excessive dispersion and separation. For substances that do not contain biodegradable substances to be tested or other testing reagents, the surface of the paper must have good optical properties (such as strong light permeability) to facilitate the reproduction, modification and generation of signals in the subsequent process and minimize interference with the analysis signals. There are many types of paper, where 30% to 90% of the paper is pores, and the pore shape in the paper are not the same, and thus the pore structure of paper allows it to play a special role in different fields. The nitrifying part of the fiber can also be prepared into nitrifying fibers. Nitrification enhances the porosity of cellulose and changes it from hydrophilic to hydrophobic. Due to the working principle of paper chips, at present, microfluidic paper chips need paper with strong water absorption, and thus filter paper with good absorption performance and large pores is adopted as the base material of paper chips, which is considered the best matrix material. However, there are many types of filter paper, and different types should be selected according to different applications. Other paper types will also pass detection requirements. When filtering blood cells in whole blood analysis, because blood cells are prone to deform through filter paper with pores larger or slightly smaller than their diameter, Whatman grade 1 filter paper with low porosity, small pore size and uniform distribution should be selected.^28^ Due to its good non-specific adsorption of biomolecules, nitrocellulose is suitable for fixing biomolecules such as proteins and DNA (Table 2).^29^

**Table 2:** Paper types and corresponding characteristics

Filter paper type	Characteristics	The applicable objects
Whatman1 qualitative filter paper	The surface is smooth and uniform, with small interstitial spaces, and the liquid flows at a medium speed on its surface	Suitable for the filtration of easily deformed blood cells
Whatman4 qualitative filter paper	With larger filter hole and grain retention, the liquid can flow faster on its surface	—
Whatman 3 MM tomographic paper	High purity and consistency	It can be used in the case of capillary effect, uniform load and wide application area
Grade1 international standard chromatographic paper	—	For normal analysis separation
Cellulose nitrate film (NC film)	Nitrocellulose has good nonspecific adsorption to biomolecules	Fix biomolecules like proteins or DNA
A paper towel	Good water absorption and easy to obtain	Fixed hemocyte
Glossy paper	Good toughness, low degradation, smooth surface	The nanoparticle can be fixed on its surface
Fiberglass paper	It is conducive to the grafting of quantum dots	Used for detecting metal ions with quantum dot ion imprinting technology

### 5.4 Features of paper chips

#### 5.4.1 Their processing cost is low

Compared with the base silica gel, glass and polymer of traditional microfluidic technology, the base paper of microfluidic paper chip has abundant sources, and because the price of paper is much lower than the above-mentioned materials, the processing cost is greatly reduced. The production of two-dimensional paper chips is completed *via* photoelectric printing, waxy printing, inkjet printing, electronic drawing, *etc.* Three-dimensional paper chips can be made by folding and stacking two-dimensional paper chips, which not only greatly reduces the production difficulty, but also the production cost. Compared with traditional microfluidic paper chips, microfluidic paper chips have great cost advantages.

#### 5.4.2 The analysis system should be portable

Compared with silica gel, glass and polymer, the substrate of microfluidic paper chip has capillary force. The paper is then patterned with hydrophobic treatment so that the solution can be directed in an orderly manner. The thickness of paper is moderate, usually dozens to hundreds of microns, and thus it is light and easy to fold, and easy to carry and transport.

#### 5.4.3 Good biocompatibility

Paper chip paper mainly uses different properties of filter paper, which is mainly composed of cellulose. Because cellulose itself has good biocompatibility and paper has a large specific surface area, proteins, enzymes, DNA and other biological macromolecules can be fixed on its surface. Cellulose is insoluble in organic solvents, which gives paper excellent chemical stability, and paper has chemical inertia, and thus can be used as a reaction carrier in chemistry, biochemistry and medical research fields.

#### 5.4.4 Follow-up green treatment

Considering that the main substrate of paper chips is paper, the subsequent treatment is very simple, does not cause any pollution and is non-toxic and biodegradable. Generally, paper chips are disposable analysis equipment, especially in remote and underdeveloped areas. After the paper chips are used, they only need to be processed in a simple way without affecting the environment.

#### 5.4.5 No driving device required

Albert Folch, an Associate Professor of Bioengineering at the University of Washington, once said “the water absorption of paper is the cheapest suction pump on earth.” Due to the capillary action of paper, paper chips using paper as the base can drive the analysis sample without any external force to drive the pump, and thus liquid vector flow. Also, the cellulose in filter paper is a reticulated structure, which can avoid the disadvantage of bubbles easily generated when an external drive pump is used in the traditional microfluidic chip.

#### 5.4.6 The detection effect is obvious

In paper chips, filter paper is usually used as the base material, which is mostly white with a low detection background, and thus has an obvious contrast effect in any detection and color development reaction. Accordingly, it can be used as an excellent material for parallax detection, and has advantages in carrying out colorimetric analysis, which is also beneficial for photometric detection.

#### 5.4.7 It is plastic

Paper has strong ability to become plastic because its fiber surface contains a large number of hydroxyls and a small amount of carboxyls, and thus chemical modification of its surface is easily carried out. Also, paper can be easily processed into a complex three-dimensional structure considering that folding and bending will not make it break. Thus, paper is flexible and suitable for printing.^30^

#### 5.4.8 Strict transportation conditions

The long-distance transportation of enzymes, antibodies, and antigens require strictly controlled storage conditions because temperature and humidity affect the flow rate of fluids and the specific recognition between molecules. Also, paper chips tend to break easily, making them unsuitable for heating or long soaking.

#### 5.4.9 Different subjective judgments

In particular, in colorimetric detection, various results will appear by naked eye observation, resulting in different subjective judgments and explanations. Also, settings may be controversial, especially when the detection signal is close to the critical value.

### 5.5 Paper chip liquid flow rate control

#### 5.5.1 Slowing down liquid flow control

There are two methods to slow down the flow of liquid on paper chips: (1) slowing down the flow rate by changing the geometry of the paper channel^31,32^ and (2) by changing the pore radius of the porous media in the hydrophilic channel through the deposition of chemical substances in the paper channel, the flow velocity is slowed down.^33,34^

#### 5.5.2 Speeding up liquid flow control

The main method to slow down the liquid flow velocity on paper chips is to increase the porous medium radius indirectly, thus speeding up the flow velocity.^35^

## 6 Paper chip production principle

The production principle of paper chips can be roughly divided into three types: (1) paper thin channel physical obstruction. Capillary channel blocking on paper does not involve any chemical reaction between hydrophobic substances and cellulose, but mainly involves the addition of reagents into the thin channels of the paper, and the presence of these reagents changes the wettability of the paper surface to form different hydrophilic and hydrophobic regions. (2) Physical sedimentation of hydrophobic reagents on paper surface. The physical deposition of hydrophobic reagents also does not involve any chemical reaction between hydrophobic substances and cellulose fibers, mainly because the added reagents deposited on the paper surface change the wettability of the paper surface so as to form different hydrophilic and hydrophobic regions. (3) Chemical modification of paper surface. Chemical modification involves the reaction between the added reagent and cellulose hydroxyl (–OH) to modify the paper to achieve surface hydrophilic and hydrophobic paper.

## 7 Production method of two-dimensional paper chips

The principle for the fabrication of two-dimensional paper chips is to confine and guide the fluid by forming channels with hydrophobic materials that can solidify. These materials include wax, polydimethylsiloxane (PDMS), SU-8, polystyrene, alkyl ketene dimer (AKD), and polymethacrylamide (PoNBMA). There are two main categories, the two-step method and one-step method. The two-step method mainly uses physical modification or chemical modification (such as physical deposition, physical blockage and chemical bonding) to make the entire piece of paper uniformly modified by hydrophobic materials, and then makes a local area dehydrated through a series of technologies. These technologies mainly include the use of ultraviolet lithography, plasma treatment, ink-jet solvent etching and other technical means of local dehydrating. The one-step method is mainly directly in the hydrophilic paper local area once hydrophobic treatment or directly in the hydrophobic paper local area once hydrophilic treatment. It mainly includes the application of wax printing, inkjet printing, drawing, screen printing, flexo printing and laser etching to achieve local hydrophobic or hydrophilic treatment.

### 7.1 Two-step method

#### 7.1.1 UV lithography

UV lithography^36–43^ first involves physical modification or chemical modification (such as physical deposition and physical blockage or chemical bonding), changing the photosensitive reagent on the filter paper, and then through a certain way protecting a specific area from ultraviolet light irradiation. The exposed area contains a photosensitive polymerization or light degradation reagent, thus forming a channel network on the filter paper. Although this method is one of the most widely used methods for making paper chips, the photoresist used in this technology is not only toxic but also difficult, which leads to the formation of hydrophobic areas that are particularly easy to bend and fold, and thus easily destroyed. Moreover, it is expensive, the operation is complicated, and the requirements for environmental cleanliness are very high.

#### 7.1.2 Plasma processing technology

Plasma processing technology^44–51^ is the use of a type of commonly used synthetic sizing agent alkyl ketene dimer (AKD) to react with the hydroxyl groups in the cellulose of paper, and chemical combination on the surface of fiber, making the paper hydrophobic. The advantage of this method is that the whole method is inexpensive to the low price of AKD. However, the disadvantage is that in the plasma treatment process, the plasma atmosphere will leak from the hollow area to the covering area, resulting in unnecessary hydrophilic or hydrophobic areas, thus reducing the accuracy.

#### 7.1.3 Etching with ink jet solution

Ink jet solution etching^52,53^ mainly involves soaking the selected filter paper in a certain concentration of polystyrene solution for a period, and then waiting for the solution to evaporate completely before the hydrophilic filter paper becomes hydrophobic. At this point, multiple toluene solutions are printed with a printer in the selected area to remove the polystyrene and restore its original hydrophilicity to form a hydrophilic channel. The advantage of this method is that it requires only a printing device to fix the reaction reagent directly on the paper-based material. However, the complex multiple printing steps lead to low production, and thus high-volume production is difficult to achieve.

### 7.2 One-step method

#### 7.2.1 Screen printing technology

Screen printing technology^54–59^ is a widely used printing technology in printing plants. This method was later applied to the production of microfluidic paper chip technology. Firstly, the desired shape of the plate is made, and then the heated solid wax is placed on the plate and heated again, so that all the solid wax melts and penetrates into the plate base hole to form a hydrophobic barrier. The advantage of this method is that it is easy to operate and can be used for mass production. However, its resolution is relatively low and the shape of the net needs to be customized, which has limitations on the pattern shape produced.

#### 7.2.2 Flexo printing technology

Flexo printing technology^60^ is a printing method for printing inks with flexible reticulation. Adagio printing is the use of tools to evenly coat a certain thickness of ink on the printing plate raised by a specific part of the pattern of the ink on the surface of the imprint under the pressure of the roller. This method not only requires a set of specialized equipment and the plate-making process is complex, but also requires highly smooth paper.

#### 7.2.3 Drawing technology

##### 7.2.3.1 Automatic plotter plotting

Polydimethylsiloxane (PDMS) is a common substrate for conventional microfluidic chips. Plotter automatic cartography^61,62^ is mainly controlled by a computer program, which has an ink plotter with a very dilute solution of PDMS, and on the filter paper, selected areas depict the pattern of the design. After PDMS is cured in the region of the filter paper selected, it forms hydrophobic DAMS, and the other areas still maintain their original hydrophilic channel, thus resulting in the formation of paper chips. The advantages of this method are that the paper chip is more flexible and its cost is relatively low. However, the resolution of the hydrophilic channel and hydrophobic channel is low.

##### 7.2.3.2 Manual drawing with oil-based pen

The manual drawing method using an oily pen involves placing the mould of the desired shape above the filter paper, use an oily marker or crayon to draw, and wait for the oily pen and oil to air dry to form a hydrophobic barrier. The advantages of this method are that the operation is extremely simple, convenient and fast, the paper chip produced is flexible, no complex instruments and related professionals are required, and the cost is very low. However, its disadvantage is that it is difficult to operate in the case of complex patterns and the resolution is very low.

#### 7.2.4 Cutting method

##### 7.2.4.1 Manual cutting

Manual cutting^63,64^ is mainly used to achieve cutting on various types of paper-based materials to form different types of two-dimensional porous sheets by manually using a knife and comparing the required patterns. However, the resolution of this method is extremely low.

##### 7.2.4.2 Craft knife cutting method

The craft knife cutting method in ref. 65 and 66 involve the use of a computer-controlled cutting knife that can rotate freely such as an *x*–*y* plotter knife and adjusting the blade angle and the appropriate force to realize accurate cutting on various types of paper base material (including small radius curve) and formation of different types of porous thin two-dimensional space, thus creating paper chips. The advantages of this method are that compared with the drawing technique, the traditional ink or oil-based pen is replaced by a cutting knife, which overcomes the difficulty of operation in the case of complex patterns, and different types of complex microfluidic channels can be cut. However, it is usually necessary to put a protective layer on the bottom to prevent the filter paper from being cut.

##### 7.2.4.3 Laser cutting technology

Laser cutting technology^67–69^ is applicable for the production of paper chips from hydrophobic paper. Compared with other methods, the resulting paper chips have a higher resolution and do not need a protective layer at the bottom compared with the knife cutting method. However, the preparation cost is high and the channel needs to be modified with nanoparticles to drive the liquid flow. Also, the specialized laser cutting equipment required, which is expensive.

#### 7.2.5 Printing method

##### 7.2.5.1 Inkjet printing technology

Inkjet printing^52,53,70–75^ is a method of printing a hydrophobic reagent on a paper substrate to make a paper chip. Specifically, a specific printer is used to print a hydrophobic reagent on local filter paper, and then a series of physical and chemical changes are generated inside the filter paper after heating at a certain temperature. Consequently, the hydrophobic reagent is combined with the filter paper to show hydrophobic characteristics, while the place without printing is still hydrophilic, thus forming a paper chip. The advantage of this method is that it does not require the assistance of a mold and the operation is relatively simple, which can be used for mass production quickly. However, the need for improved printers for accurate measurements makes specialized inkjet printers expensive and difficult to buy. Andres' team^25^ of Harvard University used solid wax to construct hydrophilic and hydrophobic channels and built a visible colorimetric plate by inkjet printing in the detection area. A simple colorimetric method was applied to build a paper-based chip.

##### 7.2.5.2 Wax spray printing method

Wax printing technology^13,76–96^ involves the use of hand-drawn graphics, crayon copy-printed graphics and wax printing method in a particular area of filter paper, and three methods will form a solid wax pattern. Subsequently, the filter paper is placed in a high temperature furnace (150 °C), the solid wax melts and infiltrates the filter paper to form a thin dam, and at that moment, paper chips are formed with millimeter-sized hydrophilic channels. This method can realize rapid batch production, but the obtained pattern resolution is not high.

##### 7.2.5.3 Laser printing method (LP)

Laser printing (LP)^97^ has received extensive attention in the manufacturing process. This method mainly involves the use of carbon powder particles to form a dry thermoplastic polymer powder, which is mixed with carbon black or colorant, that is, during the development process by electrostatic selective collection, and then transferred to the paper. The transferred pattern is then heated to form a permanent image on the paper during fusion with a molten thermoplastic polymer powder. The advantage of this approach is that the whole printing process is totally solvent free and highly efficient. However, due to the addition of conductive powder to the toner directly, electrostatic leakage is caused, which leads to the failure of the toner in the transportation process. Consequently, the printed circuit cannot use the standard LP process.

#### 7.2.6 Wax melting and soaking technology

Melting wax soaked technology^98–100^ will have specific shape metal mold in the top, a filter paper and then a magnet in the bottom of the glass slide. The paper is clipped in the middle of the device and heated to 140–160 °C after the addition of liquid BW, BW is removed after cooling and solidification. Then the mold area is covered to keep the original hydrophilic paper, wax and other exposed area due to the penetration, thus creating hydrophobic chips. The advantages of this method are low reagent cost and simple procedure. However, due to the poor reproducibility and need for metal molds of a particular shape that are not readily available, mass production is not possible.

#### 7.2.7 Elastic printing technology

Elastic printing technology^101^ mainly involves printing styrene solution dissolved in a volatile solvent on paper to prepare a hydrophobic area, and then using the viscosity of the solvent and steam pressure to permeate styrene into the paper fiber to form a hydrophobic channel, thus creating paper chips.

#### 7.2.8 Chemical vapor deposition

Using chemical vapor deposition,^102,103^ paper is placed between metal molds and magnets to cause the hydrophobic monomers to evaporate in the sublimation chamber. After pyrolysis, hydrophobic areas are formed in areas not covered by metal molds due to polymerization and deposition, and thus paper chips are formed (Table 3).

**Table 3:** Production methods of two-dimensional paper chips

	Technical means		Hydrophobic reagent	Resolution	Principle of combination	Advantages	Disadvantages
One step	Printing method	Inkjet printing technology	AKD	High	Chemically bonded	The reagent is cheap, the procedure is simple, and can realize fast batch production	The cost is high and the printer needs to be modified to be more accurate
		Spray wax printing technology	Wax	Low	Physical plugging	Simple steps, low cost, and no mold and chemical reagents required	The instruments are expensive and the chips are unstable at high temperatures
			PDMS	Low	Physical plugging	Low reagent	The pattern resolution is not high
		Laser printing (LP)	Solid powdered ink	Medium	Physical deposition	The whole printing process is highly efficient and totally solvent free	Adding conductive powder directly to toner will cause electrostatic leakage and transfer failure. Therefore, the standard LP process cannot be used for printed circuits.
Melt wax soaking technique			Wax	Medium	Physical plugging	The steps are simple and the cost is low	Metal mold is not easy to obtain, poor reproducibility, and no batch production
Flexographic printing technology			Styrene	Medium	Physical deposition	—	—
Chemical vapor deposition			Hydrophobic monomer	Medium	Physical deposition	—	Metal molds are not readily available
Two step	Ultraviolet lithography		SU-8	High	Physical plugging	The cost is low	The reagents are expensive, complex, the resulting chip is not bendable and folded, and cross-contamination occurs easily
			OTS	High	Chemically bonded	No organic solvents are required and malleable	Expensive instrument required
			PoNBMA	High	Physical deposition	Reagents are readily available and cheap	The instrument is expensive and does not fold
	Plasma processing technology		AKD	High	Chemically bonded	Simple steps and cheap reagents	The instrument is expensive, need to custom make metal mask separately and plasma atmosphere is easy to leak
	Ink-jet solution etch		Polystyrene	Medium	Physical deposition	Fix the reagents directly	Multiple printing, cumbersome operation, and no batch production
One step	Screen printing technology		Wax	Medium	Physical plugging	Simple operation, can be mass production	There are limits to making patterns
	Flexo printing technology		Polystyrene	Low	Physical deposition	The steps are simple and can be produced in batches	The operation is complicated and affected by the smoothness of the paper
			PDMS	Low	Physical plugging	The steps are simple and can be produced in batches	Affected by paper quality
	The drawing technology	Automatic pl-otter plotting	PDMS	Low	Physical plugging	The reagent is cheap and keeps the filter paper flexible	It is difficult to draw complex patterns
		Manual drawing with oil-based pen	Wax	Low	Physical plugging	The method is simple and requires no professional	It is difficult to draw complex patterns
			Oily ink	Low	Physical deposition	The method is simple and requires no professional	It is difficult to draw complex patterns
	Cutting method	Manual cutting	No	Very low	No	Low cost, no chemical reagents required	It is difficult to draw complex patterns
		Process knife cutting method	No	Low	No	Low cost, no chemical reagents required	Cover to prevent contamination and add a protective layer at the bottom to prevent the filter paper from being cut
		Laser cutting technique	Arbitrary hydrophobic paper	High	No	Low cost, no chemical reagents, and no protective coating at the bottom	Channels need to be modified with nanoparticles

## 8 Production method of 3D paper chips

Three-dimensional paper chips are made by stacking two-dimensional paper chips on the *Z* axis. 3D paper chips possess some better properties than 2D paper chips because of their three-dimensional channel network structure, such as the rapid transfer of fluid, high-throughput detection in a monolithic device, and easy to design functional components.

### 8.1 Superposition method

The stacking method^104^ mainly involves making a two-dimensional paper chip with two-dimensional production technology, and then using some special means to align the two-dimensional paper chips, glue and stack them together, making a three-dimensional paper chip. The advantage of this method is that the fluid can flow in the vertical direction compared with the two-dimensional paper chip. However, this method is time-consuming and requires accurate alignment between layers.

### 8.2 Origami method

Origami is a new way of making paper chips using traditional origami techniques.^105^ A simple technique is used to modify the channel, liquid storage tank, crease and other analysis elements on the paper chip, and then fold the paper chip into a multi-layer device according to the crease and specific folding method, thus producing a 3D paper chip waiting for sample injection. The advantage of this method is that compared with the superposition method, the process is simple and fast, only one paper chip needs to be modified, and each layer can be used as the detection layer because the 3D paper chip can be folded again. However, the disadvantage is that additional metal splints are required.

### 8.3 Glue spraying method

The spraying method^106^ mainly refers to spray the binder evenly on the other side of the prepared sheet-fed chip, and then gluing the processed sheet-fed chip together in accordance with the design order to make a 3D paper chip.

### 8.4 Double-sided adhesive bonding method

The double-sided adhesive bonding method^40,45,107–109^ involves cutting special double-sided adhesive tapes into the same shape, and then bonding them with paper chips cut in the same shape to make three-dimensional paper chips.

### 8.5 Stapler binding method

The stapler binding method^110^ involves stacking the paper chips together after they are made, and then binding them with a stapler, which is used daily. The advantages of this method are that it is simple to operate, does not require complex instruments, and is inexpensive. Moreover, the production process is only printing and binding, which can be increased in the same proportion. Also, the flow rate is faster than that from the double-sided adhesive tape and origami methods. However, once the paper chips are all bound, their position cannot be changed, and thus the flexibility is relatively poor.

### 8.6 Ion imprinting technique

**Fig. 2:**
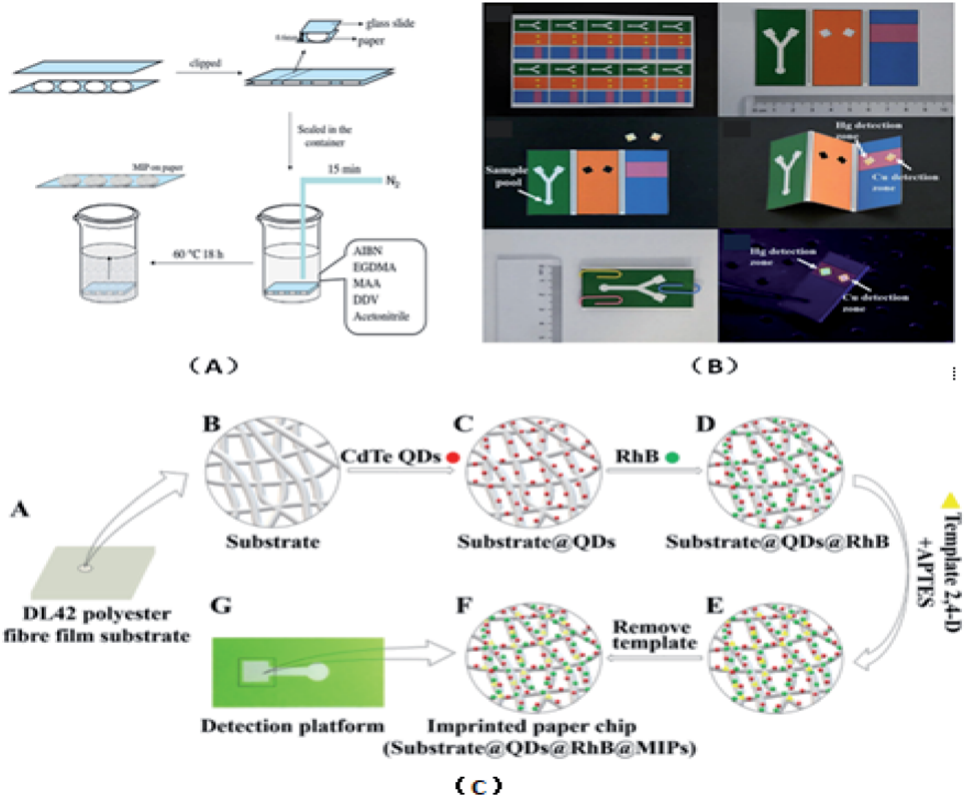
(A) Schematic diagram for the preparation of molecularly imprinted polymer on DDV paper. (B) Paper chip of 3D quantum dot molecular imprinting technology for the detection of Cu^2+^ and Hg^2+^. (C) Synthesis of detection platform.

The ion-imprinting technique^111–114^ is mainly used to make quantum dots with different emission wavelengths form carboxyl groups with cellulose, which are firmly grafted on the paper base due to covalent bonding, and then grafted with different ion-imprinted polymers on the surface of two glass fiber paper bases. Fig. 2(A) shows is a schematic of the preparation of a molecularly imprinted polymer (MIP) on paper. As shown in Fig. 2(B), the chip is composed of three layers. The top layer has a hydrophilic “Y”-shaped channel, which is used to deliver the sample to the testing pool through the capillary force of the paper. The middle layer is pre-waxed and then two square cavities are cut out with a knife. The bottom layer is a base that is patterned entirely with wax to support the paper chip components and resist liquids. At this point, a 3D quantum-dot ion-imprinted polymer paper chip is formed. The advantage of this method is the existence of the ion imprinting layer not only can recognize and combine with the template ion, but also stop interference ions to ensure the fluorescence from ion disruption of the quantum dots and quenching. Also, due to the covalent bond effect, which allows quantum dot solids to be grafted on paper and make the signal is more stable, and the formation of the three-dimensional paper chips after joining the template ion fluorescence quenching signal, the fluorescent signal can be recovered after elution of the template ion. However, because silica is the main component on the surface of glass fiber, there are obvious impurities, and thus the pretreatment should be done first, and this preparation method can only be combined with the fluorescence detection method. Using molecular imprinting technology, Guoying Hao's group^115^ developed a multifunctional rapid ratio fluorescence sensing platform. The dual fluorescence signals in the platform make the color changes generated by ultraviolet light more accurate, and the operation is simple, inexpensive, there is no need for professional personnel, and the template is quickly and specifically detected and recognized. Thus, has been used in on-site home therapy and commercial products, facilitating the further development of POCT. As shown in Fig. 2(C), A is the DL42 substrate and B–F show the substrate treatment of the imprinted paper chip, the graft of two fluorescent substances and the formation of molecular imprinted recognition sites. G is the final product of the analyte that is actually tested. Among them, APTES is a functional monomer, RHB is a reference fluorescent substance, and 2,4-D is a template molecule for the analytical chemical sensing platform. The cross-linking agent TEOS is also required (Table 4).

**Table 4:** Production methods for 3D paper chips

Technical means	Design is difficult	Aim at easy	Speed	Operation skills	Cascading way	Advantages	Disadvantages
Superposition method	General	More difficult	Medium	Medium	Multiple	The fluid can flow in the vertical direction	The steps are tedious and the layers need to be aligned accurately
Origami method	Complex	Simple	Medium	Medium	One	The steps are simple and fast, and each layer can be used as a detection layer	Additional metal splints are needed
Spray glue method	General	Difficult	Slow	High	One	—	—
Double-sided adhesive bonding method	General	More difficult	Slow	High	One	—	—
Stapler binding	Simple	General	Fast	Medium	One	Simple operation, low cost, and can be the same proportion of the increase	The flexibility is relatively poor
Ion imprinting technique	General	General	Fast	Medium	One	Blocking interference ions to prevent the fluorescence quenching of quantum dots	Preprocessing should be done first, and the detection method has limitations

## 9 Paper chip detection method

Paper chip detection means are diverse, which provide convenient conditions for analysis and detection, but which detection method to choose to detect the substance to be tested is determined by the nature of the signal substance.

### 9.1 Colorimetric test method

Colorimetric detection^49,50,60,70,76,77,80,84,92,95,99,116–126^ is the most commonly used detection method in paper chip analysis. This involves a chromogenic reaction when the sample solution in the area of measurement, the target material and reagent reaction cause the components to be detected as a colored material using the contrast color depth and relevance of the component concentration under test, quantitative or semi-quantitative analysis, and exploiting the specificity of the reaction to the guarantee system of higher selectivity. This type of detection can be identified directly by the naked eye, thus allowing semi-quantitative analysis. Some electronic products (such as digital cameras, smart phones and scanners) can also be used to scan the color changes after the experiment into images, and then Photoshop, Image J and other image processing software can be used to convert the image color into a gray value. Finally, quantitative analysis can be conducted according to the relationship between the gray value and the concentration of the object to be measured. Compared with the traditional detection method, this method has the advantages of being fast, simple, low cost and highly selective.

### 9.2 Electrochemical detection

Among the electrochemical detection methods, the ampere method is the most widely used one, in addition to the potential method, volt ampere method and conductance method.

Electrochemical detection^66,71,77,89,100,101,104,127^ is a method by which the chemical signals of the components to be measured are converted into electrical signals by an electrical sensor. Compared with the colorimetric detection method, this method has the advantages of stable signal, high sensitivity, good selectivity, good accuracy, easy integration and miniaturization. Compared with the optical detection method, this method has less background interference and higher sensitivity. However, its disadvantage is that it increases the complexity of equipment and costs, the detection pool needs special design, and the detection substance needs electrochemical activity, requiring full chemical reaction.

#### 9.2.1 Ampere detection

Amperometric testing was first proposed by Wooley *et al.* in 1998. Amperometry monitors electroactive substances by measuring synthetic currents. Due to the potential difference between the solution of the tested object and the electrode, when the solution of the tested object flows through the surface of the working electrode, the electrically active object to be tested will gain (lose) electrons, and thus a redox reaction will occur. The innovation point is to apply a constant electric potential to the working electrode. However, this method produces interference under the separation voltage of capillary electrophoresis, and thus to isolate the three electric fields, three detection strategies should be employed, namely, in channel, out of channel, and channel edge.

#### 9.2.2 Conductivity detection

Conductance detection is a quantitative analysis of the difference between the bulk solution and the analyte region by detecting the direct contact between the electrode and electrolyte solution. However, this method has large background interference and is prone to bubble generation, and thus the two electrodes should be placed outside the separation channel for the non-contact conductance detection of capillary electrophoresis to reduce background noise and avoid the generation of bubbles.

#### 9.2.3 Electric potential detection

According to the research by Walter *et al.*^128^ in 2004, potential detection mainly uses the potential difference between the ionic activities of different solutions on both sides of the membrane to detect the potential. When analyzing the flow of a semi-permeable membrane through an ion-selective electrode, there is a potential difference between the external solution and the internal solution. The advantage of this method is that it is specific because it needs to be based on an ion-selective membrane. However, the limitation of this method is that when the potential detection is combined with the separation step, multiple detection of the analyte cannot be performed, and the ion-selective membrane cannot pass through the buffer ions in the background and must be a semi-permeable membrane.

### 9.3 Optical detection method

#### 9.3.1 Fluorescence detection method

The fluorescence detection method^13,18,46,63,77,82,85,86,94,95,98,112,113,129^ is mainly used in combination with microfluidic systems. It is mainly used to determine the fluorescence samples and fluorescent markers by using the fluorescence of some substances emitted by light irradiation. The laser induced fluorescence (LIF) detection system^96^ is matched with the size of the chip. Due to the low scattering of the laser beam, ideal results can be obtained with only a small volume of sample. The advantages of this method are its good selectivity, high sensitivity and low detection limit. However, the detection system is complex, the instrument is large in volume, not easy to miniaturize, and the cost is high.

#### 9.3.2 Chemiluminescence detection method (CL)

The chemiluminescence method^48,51,105,111,130^ is a type of trace analysis method to determine the content of the corresponding component in the reaction according to the total amount or intensity of chemiluminescence at a certain time. This method does not require an excitation light source and complex and expensive spectroscopic system, and has the characteristics of high sensitivity, simple equipment, wide linear range, low interference background, simple operation, easy to realize automation and rapid analysis. However, they are less selective.

#### 9.3.3 Electrochemical luminescence detection (ECL)

Electrochemiluminescence^57,58,62,64,131,132^ is a new analytical technique combining the chemiluminescence detection method with electrochemical means. Electrochemiluminescence (ECL) mainly produces some electro-active substances on the electrode surface by the electrochemical method, and the luminescence phenomenon is accompanied by the formation of excited states between these substances or between these substances and some components in the system through electron transfer, and then returns from the excited state to the ground state. This method has all the advantages of chemiluminescence and overcomes its poor selectivity. Compared with the colorimetric method, this method has a lower detection limit.

### 9.4 Mass spectrometry

Mass spectrometry^83,133^(MS) is a microanalytical method for quantitative and structural analysis by measuring the quality and strength of ions in samples. The advantages of this method are that the detection process is fast, sensitive, and it has strong qualitative ability, and more structural information can be obtained in a single analysis.

### 9.5 Surface-enhanced Raman (SERS) detection

Surf-enhanced Raman detection^6,114,134–138^ is mainly based on scattered light with different intensities generated by the activity characteristics of chemical reaction molecules to identify analytes. The phenomenon of Raman signal amplification occurs in samples that have been adsorbed on the surface of colloidal metal particles (such as silver, gold, and copper), or on the surface of crude sugar that has been adsorbed on these metals. The advantages of this method are its short detection time, nondestructive detection and high sensitivity. However, the activity and reproducibility of the SERS substrate greatly limited the application of the surface-enhanced Raman detection method in daily life.

### 9.6 Immune detection

**Fig. 3:**
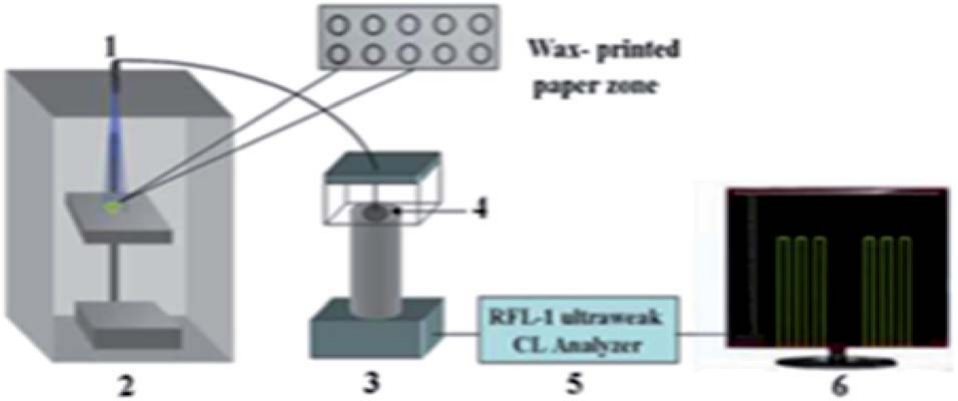
Schematic of the paper-based LIF immunoassay.

Immunoassay^22,64,98,139,140^ mainly involves the *in vitro* binding of an antibody and antigen, with the different markers of the antigen or antibody produced by specific binding can be detected by the naked eye or an instrument. The advantages of this method are its good universality and strong special separation. Wu *et al.*^140^ developed an automated, portable, integrated microfluidic system, which was used to detect influenza A with a smart phone under the POC setting. Antigen-based immunization enabled the faster, more convenient and low-cost detection of influenza A in the detection. A reagent storage module was added to the integrated paper microfluidic chip with several independent chambers. The plastic needle is punctured from the bottom to each reagent chamber so that the reagent is discharged in a certain volume and interacts with the NC membrane located on the reaction module, ensuring the sensitivity of the reaction. Zhao *et al.*^98^ set up a paper chip based on a laser-induced fluorescence immunoassay to detect alpha-fetoprotein (AFP). As shown in Fig. 3(B), it consisted of (1) a laser source with an optical fiber; (2) cassette tape; (3) detector; (4) cut-off filter on glass fiber (530 nm); and (5) ultra-weak luminescence analyzer. The method was used to linearly detect alpha-fetoprotein in the range of 0.001 ng mL^−1^ to 20.0 ng mL^−1^, and the detection limit was 0.4 pg mL^−1^. Thus, this provides a new method platform for biochemical analysis and medical clinical diagnosis of tumor biomarkers for tumor monitoring (Table 5).

**Table 5:** Paper chip detection methods

Detection technology		Basic principle	Advantages	Disadvantages
Colorimetric test		By color rendering	The operation is simple	Low sensitivity
Electrochemical detection	Ampere detection	The chemical signals in the solution to be tested are converted to electrical signals by electrodes for detection.	High sensitivity, good selectivity, small size, simple device and low cost.	The detection tank needs special design, and the detection substance needs electrochemical activity, which requires sufficient chemical reaction and poor reproducibility.
	Conductivity detection			
	Potential detection			
Optical detection method	Laser induced fluorescence detection	The intensity of fluorescence is increased by using a specific frequency of excitation light.	High sensitivity	Analysis requires the presence of fluorescence or functional groups capable of obtaining a fluorescent signal by reaction.
	Chemiluminescence assay	A method of determining the content of components in a chemical reaction by measuring the amount or intensity of light emitted during the reaction.	High sensitivity, no need for external light source, and simple equipment.	Poor selectivity, which requires a full chemical reaction.
	Electrochemical luminescence detection	A new analytical technique combining chemiluminescence detection with electrochemical means.	High sensitivity, easy to quantify, environmental light independence, and paper interference degree is low.	Chip production cost is high and operation is complex.
Mass spectrometry		A microanalytical method for quantitative and structural analysis by determining the quality and strength of ions in a sample.	It can provide the basic structure and quantitative information of biomolecules.	Affected by the interface problem between plasma and chip.
Immunoassay		Antibodies and antigens bind specifically *in vitro*.	Good universality and strong special separation.	—
Surface-enhanced Raman detection		The analyte is identified according to the scattered light with different intensities produced by the activity characteristics of the chemical reaction molecules.	Short detection time, nondestructive detection, and high sensitivity.	Application is limited by the activity and reproducibility of the SERS substrate.

## 10 Application of paper chips

The application of paper analysis can be traced back to the 20th century, among which urine analysis strips and stone steam paper are the most common diagnostic equipment of paper chips (Table 6).

**Table 6:** Application of paper chips

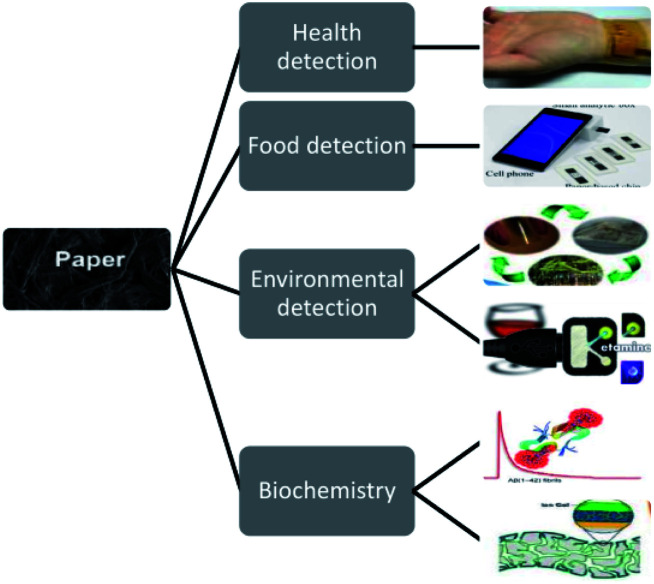

### 10.1 Health detection

The paper chip is simple to make and easy to carry, which provides a home-based method for the early warning and detection of diseases. It also provides a portable platform for detecting tumor markers in clinical diagnosis to monitor physical condition in real time.

#### 10.1.1 Drug screening

**Fig. 4:**
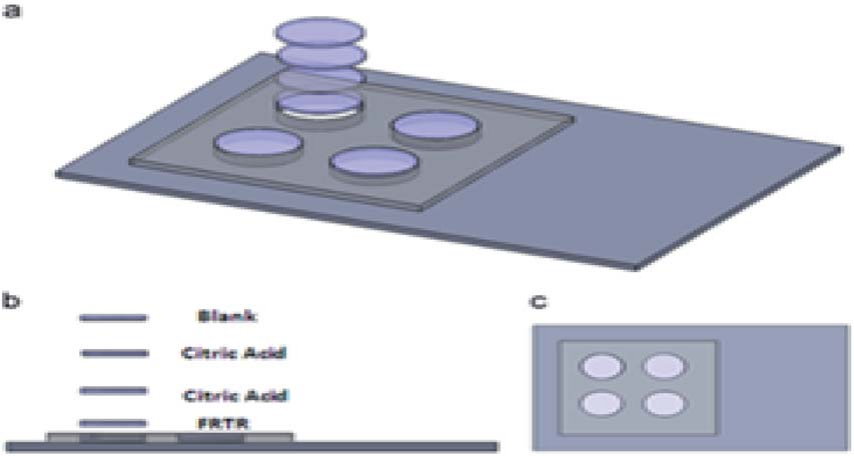
Schematic diagram of the paper test kit.

Myra T. Koesdjojo *et al.*^120^ proposed a fast, cheap and simple paper-based microfluidic-based detection kit, as shown in Fig. 4, to detect the content of artesunate in counterfeit antimalarial drugs to protect lives and prevent the development of malaria. This colorimetric analysis targets artesunate and turns yellow when the sample is added. The test can be done within minutes, and artesunate tablets can be accurately semi-quantitatively analyzed using the color analyzer on an iPhone camera to measure the health test developed on the paper chip, the food test environment, and the biochemical color intensity. The first layer is FRTR, the second and third layers are citric acid, and the fourth layer is empty and fixed to a plastic scaffold made of polymethyl methacrylate. The concentration of artesunate in this method ranged from 0.0 to 20 ng mL^−1^, and the detection limit was 0.98 ng mL^−1^. Hong's group^47^ proposed a concentration gradient generator for high-throughput drug screening. This generator was manufactured by combining paper-based microfluidic devices with cell culture microarrays. The paper-based microfluidic drug screening device consists of five layers, including two plastic fixators, two cross-linked PDMS and a paper chip in the middle.

#### 10.1.2 Diagnosis of diseases

**Fig. 5:**
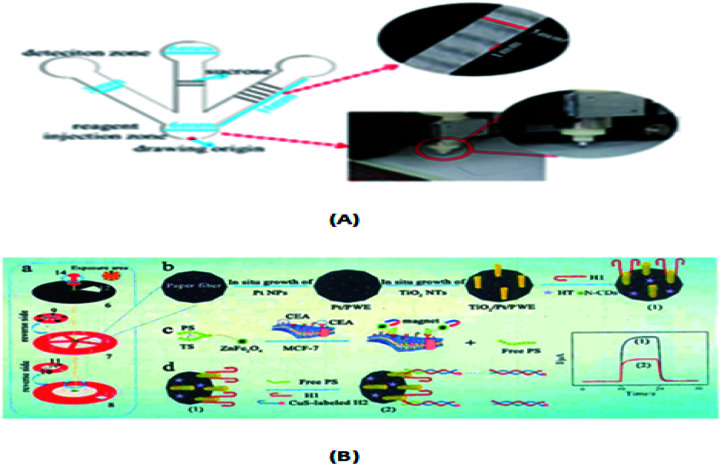
(A) Paper-based CL device and sugar drawing results. (B) (a) Addressable paper PEC chip. (b–d) Construction process for the paper-based PEC cyto-sensor.

Li's group^141^ designed a rotatable paper photoelectric controllable switch (RPPS), which was used to form a locatable paper-based photoelectric electrochemical (PEC) cell sensor for the hypersensitive detection of cell surface proteins. Activating the working area required by the cellular sensor requires a simple rotation of the RPPS, and this activation method is light selective. Hideyuki Matsuura *et al.*^82^ developed a device to quantify the clinically relevant concentration range of serum/whole blood aminoglycosides in 30–60 minutes based on a paper-based bioassay chip. Muammad S. Khan's group^135^ reported a paper-based label-free electronic biosensor chip to quantify salivary cortisol levels at the medical post (POC). The highly specific sensor chip detects cortisol at a detection limit of 3 pg mL^−1^ by conjugated antibody (anti-CAB) using 3,3′-dithiodipropionic acid di(*N*′-hydroxysuccinimide ester) (DTSP) as a self-assembled monolayer (SAM) gold (Au) microelectrode. Poly(styrene-poly(propionic acid) (PSg-b-PA22) polymer and graphene nanoplatelet (GP) suspensions were designed to be coated on filter paper to enhance the sensitivity of the immune response. Chu *et al.*^105^ developed a paper-based chemiluminescent immune device for the detection of three biomarkers, CEA, CA125 and CA199. As shown in Fig. 5(A), the precise amount of sucrose produced by a manual cutting instrument was used to create a sugar barrier on a paper-based chip microchannel to control the migration rate of reagents and the reagent transport technology, and the sensitivity of the luminol chemiluminescence system catalyzed by horseradish peroxidase was greatly improved by using gold nanoparticles as a carrier and polyhorseradish peroxidase-labeled signal antibody. Chen *et al.*^51^ established a paper-based chemiluminescence immune device combining the signal enhancement strategy of multi-enzyme carbon nanospheres for the determination of carcinoembryonic antigen in human serum. This is the first chemiluminescent immunoassay based on paper and using a multi-enzyme carbon nanosphere signal enhancement strategy. An enhanced chloride signal was observed with heat shock protein from the lumino-chlorine dioxide system loaded with polyhorseradish peroxidase. Li *et al.*^141^ also developed a visual semi-quantitative remote detection platform for human serum carcinoma embryo using the distance reading method, as shown in Fig. 5(B). It consisted of (1–5) five workspaces, (6) RPPS, (7) working sheet, (8) reference sheet, (9) working electrode, (10) reference electrode, (11) electrode, (12) scallop, (13) square hole, and (14) pushpin as the axis of rotation. The range reading method can successfully detect the level of carcinoembryonic antigen as low as 5 ng mL^−1^ and the detection limit it up to 2 ng mL^−1^.

**Fig. 6:**
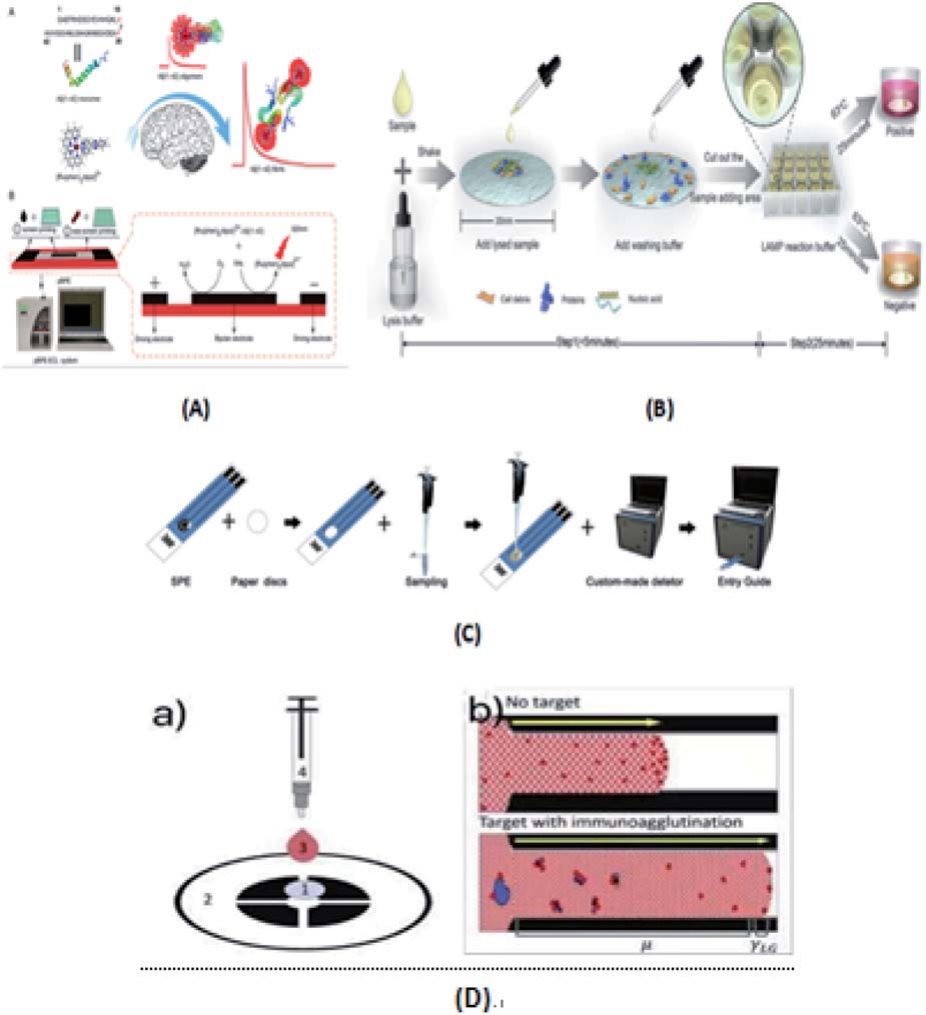
(A) Paper-based bipolar electrochemical luminescence sensor for high sensitivity Aβ(1–42) aggregation detection. (B) Paper-based LAMP method for the extraction, amplification, and naked eye readout of rotavirus at nursing point diagnosis. (C) Schematic diagram of the fabrication of the paper-based SPE. (D) Dual-layer paper chip assay procedure and schematic of the flow-based detection.

Liu's group^58^ developed an Aβ(1–42) aggregation detection method based on an unlabeled paper electrochemical luminescence sensor for the detection of amyloid protein aggregation and the potential diagnosis of Alzheimer's disease. This method is a low-cost, label-free, low-cost, sensitive one-time detection method and overcomes the limitations of traditional technologies, as shown in Fig. 6(A). Ye *et al.*^63^ reported an extremely simple paper-chip device for the field monitoring of rotavirus A, one of the most common causes of gastroenteritis in children, as shown in Fig. 6(B). The test can extract nucleic acids in less than 5 min, and the results can be immediately observed with either the naked eye or UV-vis light. Chen's group^64^ developed a new paper-based biosensor platform for ECL hepatitis B surface antigen detection. As shown in Fig. 6(C), the paper was cut into a diameter of 6 mm and laminated on the SPE. The sample was then loaded with 5 μL paper-based solid phase extraction (SPE) solution and rapidly transferred to the electrode surface by capillary action. This method can be successfully used in the clinical detection of the hepatitis B surface antigen largely due to the use of an improved maglev sandwich immunoassay, which has many advantages, such as high sensitivity, high selectivity, good reproducibility and fast analysis speed. As shown in Fig. 6(D), Tiffany-Heather Ulep *et al.*^142^ developed a double-layer paper microfluidic chip that can detect ROR1+(receptor tyrosine-like orphan receptor one) cancer cells in buffy coat samples. As shown in Fig. 6(D)(a), the first capture layer consists of a fiberglass substrate, and the second flow layer consists of a one-stage cellulose chromatographic paper with four wax-printed channels for core absorption and capillary flow-based detection. They include (1) pre-loaded red fluorescent, anti-ROR1 trapping layer (glass fiber), (2) flow layer (wax print chromatography paper), (3) erythrocyte buffy coat sample with quantified cancer cells, and (4) blunt tip syringes for uniform droplet application. As shown in Fig. 6(D)(b), they are respectively untargeted (more particles are at the flow front, increasing the interfacial tension, γLG) and targeted (more particles are not at the flow front, increasing the viscosity, μ, due to immune agglutination).

#### 10.1.3 Detection of pathogenic bacteria

**Fig. 7:**
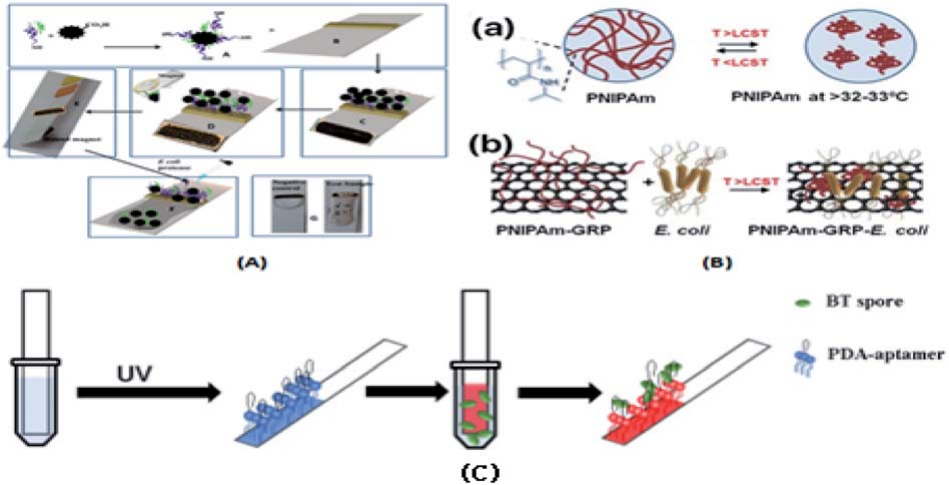
(A) Colorimetric biosensor designed to specifically detect *E. coli* O157:H7. (B, a) PNIPAm with its chemical structure in the sol state (<LCST) and in the gel state (>LCST) and (b) PNIPAm in the sol state with doped GR nanoplatelets and *E. coli*. The possibility that bacterial samples exhibit the same effect due to cellular interaction (>the LCST). (C) Schematic of the PDA-aptamer paper strip coating and the entire detection process.

Ghadeer A. R. Y *et al.*^122^ developed a simple, rapid and specific colorimetric sensor with a fixed ratio, as shown in Fig. 7(A), which can be used to detect food contamination *Escherichia coli* O157:H7 in complex foods. Muhammad S. Khan's group^143^ proposed an electrically-receptive thermally-responsive (ER-TR) sensor chip, as shown in Fig. 7(B), which was composed of simple filter paper, filter paper used as a base, and coated with poly(*N*-isopropylacrylamide) (PNIPAM) and graphene-nanoplatelet (GR) compounds. It was used to capture Gram-positive (*S. mutans* and *B. subtilis*) and Gram-negative (*E. coli*) bacterial cells in real time. Also, it was used to test the presence or absence of bacterial strains at different concentrations. Chaoge Zhou *et al.*^124^ introduced a polydiacetylene (PDA) strip sensor that can specifically identify the spores of *Bacillus thuringiensis* (BT) HD-73. This polyvinylidene fluoride (PVDF) strip was coated with a PDA vesicle that binds to a target-specific aptamer by a simple solvent evaporation method. As shown in Fig. 7(C), without additional sample preparation steps, the aptamer-modified PDA paper sensor showed a marked color shift when exposed to BT spores in solution due to the dichromate properties of polydiacetylene. Under ultraviolet light, the immobilized PDA aptamers polymerized and turned blue. After a short incubation period, the PDA aptamer recognized the BT spores and reacted, which caused the color of PDA to change from blue to red.

### 10.2 Food inspection

Food quality inspection platforms based on paper chips are not only easy to operate, but also green and pollution-free, which can play a crucial role in the inspection of food quality and the monitoring of food safety.

#### 10.2.1 Pesticide detection

**Fig. 8:**
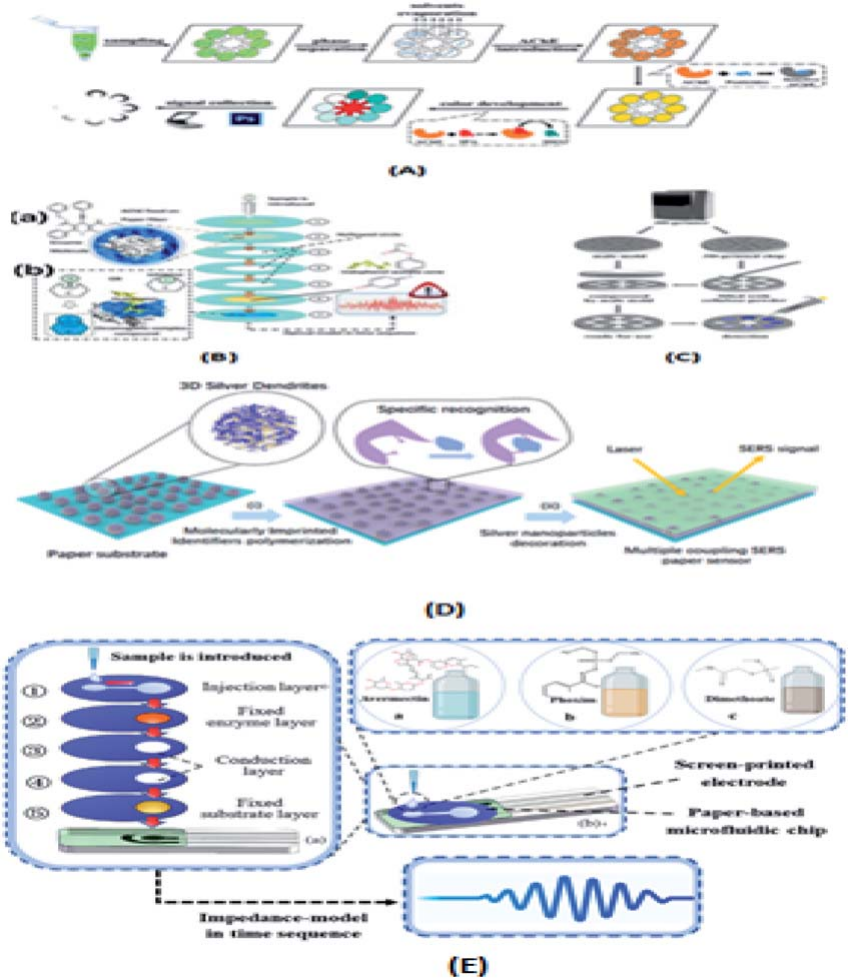
(A) Schematic of the inhibition test for food sample evaluation based on organic solvent compatible AChE. (B) Schematic diagram of the pesticide identification platform. (a) Seven-layer layout of microfluidic paper chip and molecular structure diagram of AChE and ethyl acetate (substrate). After the indophenol acetate was hydrolyzed on a chip, the absorbance was treated and analyzed by intelligent devices. (b) Chemical reaction of enzyme inhibition on the molecular scale. With the absence of inhibitor (organophosphate pesticides and carbamate pesticides), S (substrate) can be catalyzed by AChE, creating a colored complex compound, which can be introduced to the 7th layer of the proposed chip. (C) Schematic of the manufacturing process of the chip. (D) Construction of multiple coupling enhancement SERS paper substrates. (E) Schematic diagram of the OP identification platform.

Ali Turab Jafry's group^70^ used organophosphorus hydrolase to conduct environmental sensing for pesticide detection. Wei Liu *et al.*^111^ used the ion imprinting technique to detect DDV. The linear response range of this method is 3.0 ng mL^−1^ ∼1.0 μg mL^−1^, the detection limit is 0.8 ng mL^−1^, and the paper surface does not need to be activated in advance. Lili Jin's group^84^ developed a new method that omitted the enzyme prefixation program, as shown in Fig. 8(A), which improved acetylcholinesterase inhibition analysis by organic solvent extraction combined with spontaneous *in situ* solvent evaporation on the micro-fluidic paper-based analysis equipment. By adding acetylcholinesterase to the system after seven sampling steps, the *in situ* solvent evaporation process can be completed directly on the paper chip. Yang *et al.*^85^ proposed a seven-layer paper microfluidic chip, which integrates acetylcholinesterase and color reaction. As shown in Fig. 8(B), the sample is first introduced through the entrance of the first layer of manufacturing. The sample penetrates into the second layer of the chip due to the capillary force of the paper fiber. In the second layer, acetylcholinesterase can be thoroughly mixed with an aqueous solution because the three successive hollow layers allow the solution to remain in the second layer for an ideal length of time. When the sample enters the sixth layer, it is fixed with indoxyl acetate. In the presence of pesticide (inhibitor), because acetylcholinesterase activity is greatly inhibited, the incomplete hydrolysis of indole acetic acid occurs, resulting in a white or bluish circle in the pigment layer (layer 7). In the absence of an inhibitor, the indole acetate is completely hydrolyzed, producing a chemical reaction that produces acetic acid and indophenol (blue). Wang and Wu^60^ developed a portable 3D printing chip sensing system based on smart phones. As shown in Fig. 8(C), a flowered hollow channel network with a diameter of 10 mm was designed in the center of the chip, surrounded by eight straight channels with a width of 2 mm and eight detection areas with a diameter of 8 mm. The system showed a good sensing performance in the detection of organophosphorus pesticides and the analysis of many types of fruit juices. For different targets, the most effective color channel is selected for signal analysis, and the calibration equation can also be set directly in the user interface. This device can be flexibly extended to other biochemical analysis applications. Zhao *et al.*^114^ proposed a paper-based surface-enhanced Raman scattering (SERS) amplification method based on multi-layer plasmon-coupled amplification to detect neonicotinoid pesticide residues. As shown in Fig. 8(D), three-dimensional silver dendrite (SDs)/molecular identifier agent (EMIS)/silver nanoparticles (AgNPs) were used to construct SERS paper sensors. The three-dimensional dendritic 3D silver material (SDs) had a very high surface area and lightning rod effect, which was used for the first-order enhancement of paper-based sensors. Molecular recognition agents (EMIs) were coated on a reference material layer as an interlayer to capture and enrich targets. The rough surface of Ag nanoparticles (AgNPs) resonated with the local plasma, resulting in secondary enhancement. Finally, three-dimensional dendritic silver materials and silver nanoparticles were coupled to form double silver layers, and the multi-stage enhancement of SERS signals was realized under the superposition of an electromagnetic field.

Ning Yang's group^104^ proposed a method for the identification of pesticide residue in multilayer paper-based microfluidic chips with stacked screen-printed carbon electrodes (SPCE). As shown in Fig. 8(E), the microfluidic chip has five layers with a diameter of 27 mm. From the top to bottom, there is an injection layer, fixed enzyme (AchE) layer, two conduction layers and a fixed substrate (indophenol acetate) layer. Each layer has a hydrophilic zone with a diameter of 10 mm and the rest is hydrophobic. The microfluidic chip was assembled using a screen-printed electrode and double-sided tape.

#### 10.2.2 Detection of additives

**Fig. 9:**
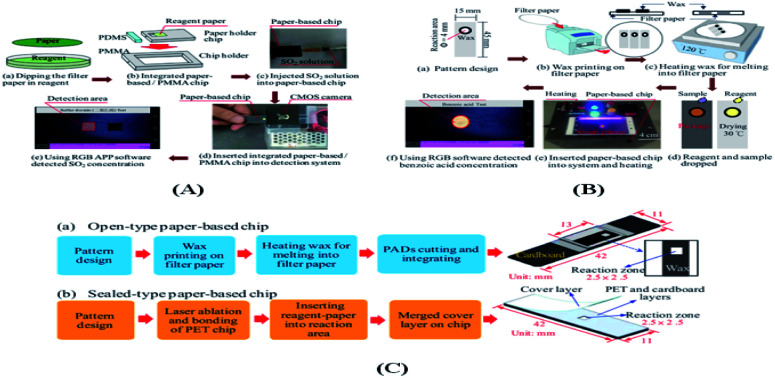
(A) Schematic illustration of microfluidic paper-based/PMMA chip and schematic illustration of associated SO_2_ con-centration detection procedure. (B) Schematic illustration showing fabrication procedure for microfluidic paper-based chip and associated benzoic acid concentration detection process. (C) Main steps in the manufacturing process: (a) open-type chip and (b) sealed-type chip.

Liu *et al.*^121^ proposed, as shown in Fig. 9(A), an integrated microfluidic platform for the detection of SO_2_ based on acid–base theory to measure the sulfur dioxide concentration in 15 commercially available food samples. Liu's group^87^ reported the fabrication of μPAD, and Fig. 9(B)(a–c) show the main steps in the fabrication process. In performing the benzoic acid detection process, as shown in Fig. 9(B)(d), 3 μL nitrification sample was dropped onto the dry pre-reaction reagent in the μPAD detection zone. As shown in Fig. 9(B)(e), the chip was then placed in the detection box and heated at a temperature of 45 °C for 20 min. The camera transmits the response image to a smartphone *via* the connector. Finally, as shown in Fig. 9(B)(f), the benzoic acid concentration was derived on the smartphone in the form of an APP. Fu *et al.*^99^ produced two types of paper chips, an open-type chip (Fig. 9(C)(a)) and a sealed-type chip (Fig. 9(C)(b)).

#### 10.2.3 Quality inspection

**Fig. 10:**
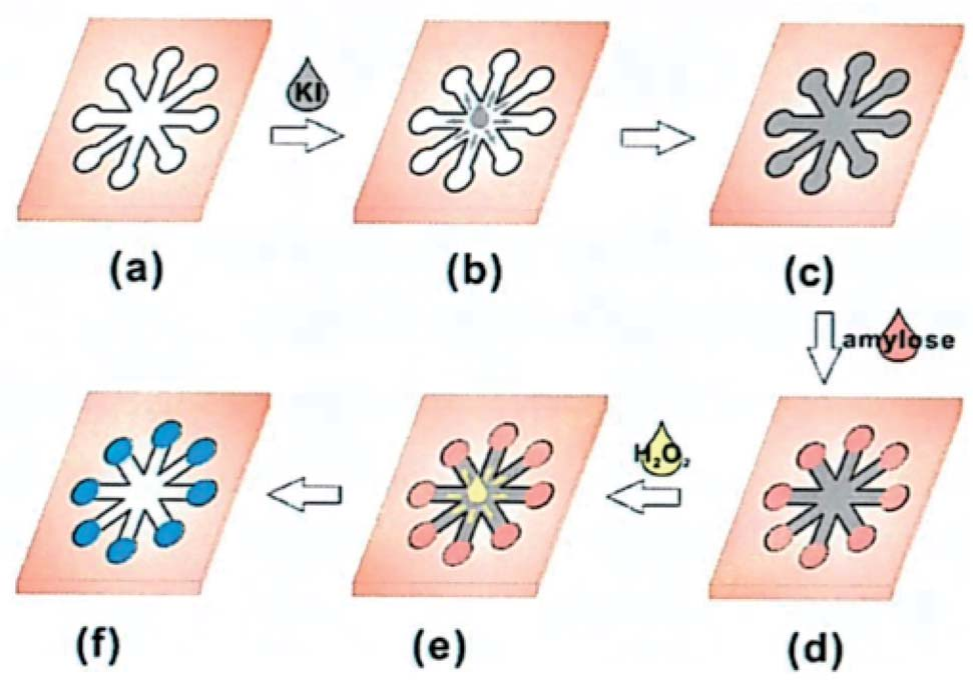
Schematic diagram of a paper-based microfiber chip process for the determination of the apparent content of amylose.

Hu *et al.*^49^ detected the amylose content in rice based on a paper chip using the color reaction between conventional starch and iodide ions. As shown in Fig. 10(a) a paper-based microfluidic chip was prepared with a network of flower-shaped hydrophilic channels; Fig. 10(b) 10 L indicator solution was transferred to the central area; Fig. 10(c) the indicator solution entered the test area through a hydrophilic channel; Fig. 10(d) 0.5 pL sample solution was delivered to 8 test areas; Fig. 10(e) 10 L of oxidant solution transferred to the central area; and Fig. 10(f) a chromogenic reaction occurred in the detection area. However, this method is not a substitute for the standard method and can only be performed in the laboratory.

### 10.3 Environmental testing

The growing environmental problems have long been a problem that cannot be ignored in daily life and in the development of national strategies. Thus, as paper chips can be employed for a variety of photoelectric analysis methods, they can be used to monitor the index of several environmental conditions. Environmental detection is not the main application of paper chips, but has become the focus of researchers.

#### 10.3.1 Heavy metal detection

**Fig. 11:**
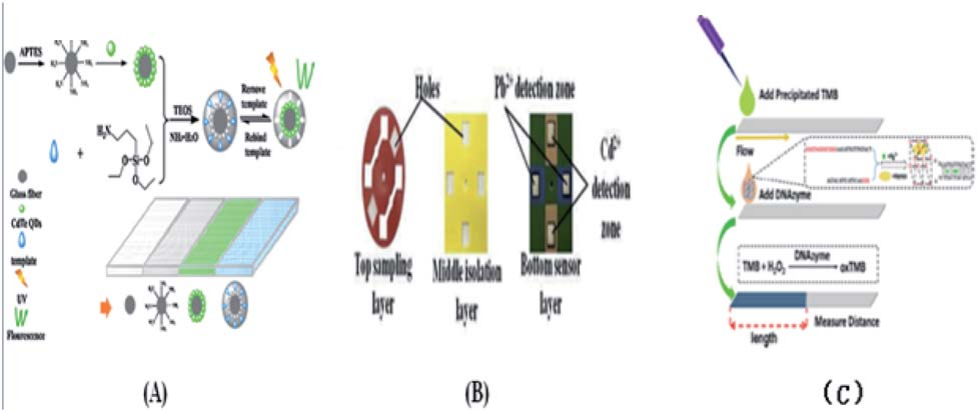
(A) Schematic illustration of the preparation and imprinting process of IIP on the surface of glass fiber paper. (B) Three-layer paper chip and its components for the specific recognition of Cd^2+^ and Pb^2+^. (C) Based on distance assay for the quantification of Hg^2+^ by G-quadruplex DNAzyme on a paper chip.

Ji Qi's group^112^ reported the synthesis of ion-imprinted polymers grafted on the surface of glass fiber paper. The preparation process is shown in Fig. 11(A). As silicon dioxide is the main component on the surface of glass fiber, there are obvious impurities. To optimize the experiment, soaking the paper components in hydrochloric acid solution can not only activate the hydroxyl group on the fiber surface, but also remove the impurities on the fiber surface. Zhou *et al.*^113^ specifically detected Cd^2+^ and Pb^2+^ using the ion imprinting technique, as shown in Fig. 11(B). Sun *et al.*^43^ proposed a portable method for the field multi-axis quantization of trace metals in air using a microfluidic paper chip. This device was combined with field colorimetric detection by smart phones and multi-axis pollution sampling of an unmanned aerial vehicle for measurement in the form of an array. The collection and quantitative use of the system for determining oxygen, copper, iron, manganese, chromium and nickel in particulate matter in the air and fly ash samples greatly reduces the analysis time and cost. The detection limits of the system for the representative metals are 8.16, 45.84, 186.0, 10.08, 152 and 80.4 ng, respectively. Liu *et al.*^145^ pre-concentrated and separated metal ions by the electrochemical detection method, with detection limits of 0.16 μg, 1.5 μg, 0.64 μg and 1.5 μg for Cr^2+^, Cu^2+^, Ni^2+^ and Co^2+^, respectively, and detection limit of 0.34 ng for Fe^3+^. Due to the presence of Hg^2+^, G-quadruplex-hemin DNAzymes formed by the hybridization of two oligonucleotides *via* T–Hg^2+^–T base pairs can bind heme to induce the catalytic activity of G-quadruplex-hemin DNAzymes. Wu *et al.*^123^ detected Hg^2+^ by the distance of Hg^2+^ mediated G-quadruplex-hemin DNAzymes formation on paper chips. The principle is that the addition of deoxyribozyme reacts with the precipitated 3,3,5,5-tetramethylbenzidine (TMB) immobilized in the sample area to produce visible and positively correlated bands of Hg^2+^ concentration. As shown in Fig. 11(C), in the presence of Hg^2+^, probes A and B can be converted to G-quadruplex DNA and then combine with heme to form DNAzyme, which reacts with the precipitated TMB to produce visible colored bands on paper. When the TMB and H_2_O_2_ concentrations are constant, the color length is proportional to the concentration of Hg^2+^.

#### 10.3.2 Component detection

**Fig. 12:**
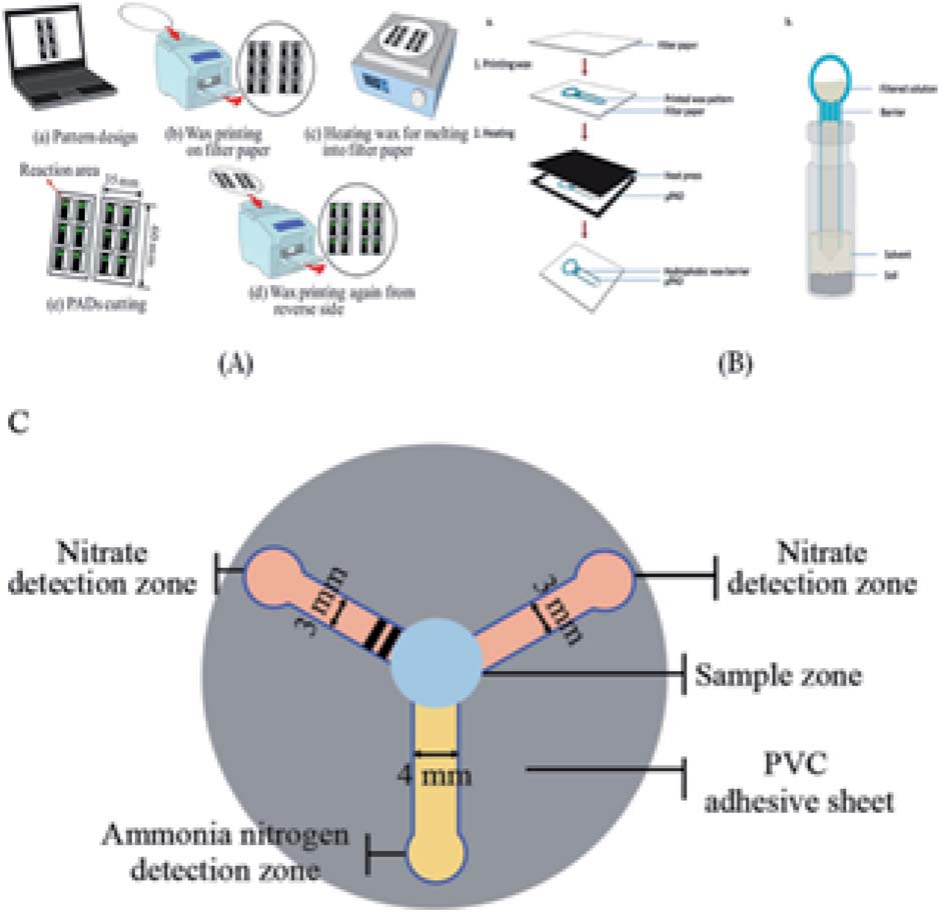
(A) Main steps in the fabrication of the designed paper-based microfluidic chip. (B, a) μPAD fabrication process involving printing the desired pattern using wax on the paper and heating it using a heat press and (b) set-up used for the extraction of explosives from soil samples. (C) Paper-based microfluidic chip structure.

Liu *et al.*^86^ introduced a method for manufacturing disposable micro-PADs. As shown in Fig. 12(A)(a), the device consists of a square reaction area with a side length of 3 mm and surrounded by impermeable wax. (Fig. 12(A)(b)). During the printing process, the equipment is placed in a furnace at 180 °C and baked for 60 s to ensure that the wax penetrates the paper thickness completely (Fig. 12(A)(c)). The PADs were cut from the paper (Fig. 12(A)(d)) and then reprinted with impermeable wax on the reverse side to prevent sample leakage (Fig. 12(A)(e)). Maiken Ueland's group^93^ reported that the device shown in Fig. 12(B) filters, extracts and pre-concentrates explosives from the soil. The detection amount of the eight explosives analyzed ranged from 1.4 ng to 5.6 ng, and the paper recovery rate ranged from 65% to 82%. Xu Rong's group^126^ developed a paper-based microfluidic chip based on color reaction for the simultaneous detection of nitrate, nitrite and ammonia nitrogen (trinitrogen) in water. The paper-based microfluidic chip contains three detection zones, three flow channels and one sampling adding area. The three detection zones are used for the detection of nitrate, nitrite and ammonia nitrogen, respectively.

### 10.4 Biochemistry

#### 10.4.1 Biological detection

**Fig. 13:**
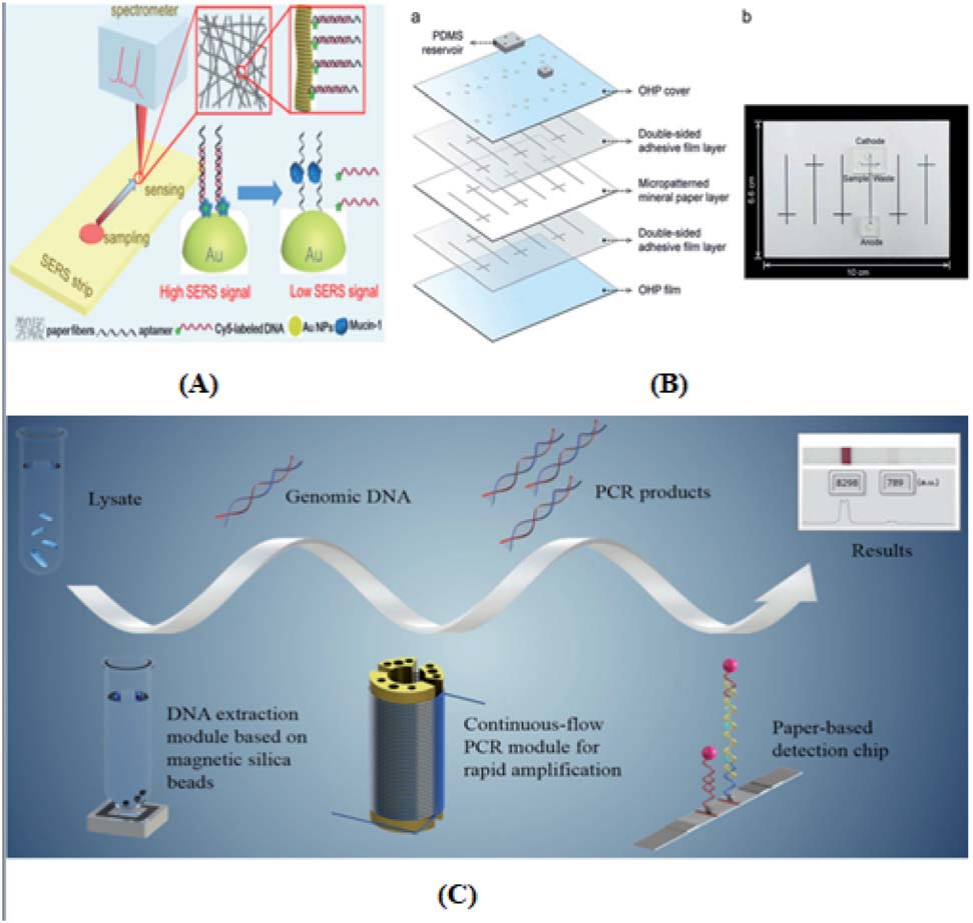
(A) Concept and design of the paper-based SERS test strip. (B, a) Six channels are used sequentially and PDMS reservoirs are placed as needed. (b) Digital image of the assembled pCE microdevice. (C) Demonstration of the operational procedure.

Fu *et al.*^88^ proposed a rapidly integrated human serum creatinine detection system, which consists of a paper chip and a detection system. During the test, the serum creatinine sample is dropped into the reaction zone of the chip, and the chip is transferred to the hot plate of the test system, where the reaction is induced by heating at 37 °C for 5 min. Lou *et al.*^42^ developed a low-cost, portable, disposable method for the rapid detection of Cl^−^ in water, which does not require complex instruments. Yu's group^45^ achieved the simultaneous separation and enrichment of protein samples containing myoglobin and cytochrome C *via* the isoelectric focusing (IEF) method. Gan *et al.*^46^ developed a low cost, rapid and automated reaction amplification microfluidic device for extracting DNA and polymerase chain from a variety of raw samples. 0.25 to 1 μL of human whole blood is subjected to DNA purification on a chip, generating 8.1 to 21.8 ng DNA, which is high than that obtained with the QIAamp@DNA trace kit. Yang's group^90^ proposed a method for the determination of the albumin concentration in human whole blood samples using a 3D microfluidic paper-based chip and intelligent detection module. The results of 40 pure albumin samples and 30 whole blood samples were consistent with that from conventional spectrophotometry (*R*2 = 0.9837, *R*2 = 0.9968). Hu's group^137^ combined the advantages of paper chips and a Raman-active substrate to develop a type of paper-based SERS strip that not only can generate condensed hot spots on the fiber when gold nanoparticles can be precisely controlled, but also enhance the size exclusion effect of the paper. Fig. 13(A) shows the decomposition diagram from the top to bottom of the printed circuit board miniature device, which is composed of six layers, including a PDMS reservoir, a contact cover, a micro-patterned mineral paper layer, a double-sided adhesive film layer and a contact film. The six channels are used sequentially and PDMS reservoirs are placed as needed.

This method overcomes the challenge of complex sample spectral sensing and achieves the quantitative detection of disease markers in whole blood. Because gold nanoparticles have multiple functions as SERS substrates for biosensors, the paper strips can be used for the quantitative detection of multiple targets. Jee Won Lee's group^66^ developed a new paper-based capillary electrophoresis (pCE) microdevice, as shown in Fig. 13(B), which is made of mineral paper and is durable, oil-resistant, tear resistant and waterproof. The entire manufacturing process can be completed in less than an hour, eliminating the need for expensive cleanroom facilities and cumbersome photolithography to separate nucleic acids, amino acids and ions. Fu's group^18^ proposed an integrated genetic analysis platform, as shown in Fig. 13(C), which can fully automate sample pretreatment, nucleic acid amplification and endpoint detection. Magnetic silica gel beads were used to separate deoxyribonucleic acid from the pyrolysis liquid to simplify the equipment design and operation. The device not only prevents cross contamination, but also greatly simplifies the operation steps and improves the detection efficiency.

#### 10.4.2 Development research

**Fig. 14:**
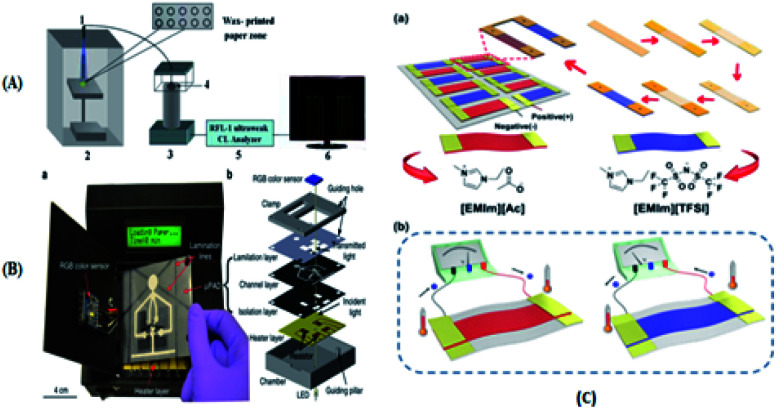
(A)Schematic diagram of the paper-based LIF immunoassay. (1) Laser source with an optical fiber; (2) cassette; (3) detector; (4) cut-off filter (530 nm) on the glass fiber; (5) ultraweak luminescence analyzer; and (6) personal computer. (B) Integration of the μPAD with a colorimetric reader. (C) Diagram of the manufacturing process and thermoelectric voltage of the ionic thermoelectric paper chip.

Chen's group^109^ proposed a paper-based microfluidic chip, which integrates nucleic acid extraction, amplification and detection for the detection of single nucleotide polymorphism. Xu *et al.*^57^ synthesized GN-HPMNS, a sensor with high efficiency, high sensitivity, good stability, high cost performance, portability and disposable use, which is more conducive to detecting compounds containing tertiary amino acids and deoxyribonucleic acid. It can also be used as an effective immobilization matrix for the preparation of solid-state sensors. Martinez *et al.*^24^ used microfluidic paper chip colorimetry to detect the content of glucose and protein. Its principle is to use glucose oxidase under the action of glucose to produce hydrogen peroxide, and the iodine ion of the chip fixed in advance in the paper will be under the action of horseradish peroxidase oxidase. The elemental hydrogen peroxide reduction of iodine due to the precipitation of iodine element in the testing area resulted in a color change from colorless to brown, thus realizing the detection of glucose. When protein samples are added, the pH value of the test area will change. Based on this characteristic, tetrabromophenol blue was selected as an indicator of acidity. When protein samples are added, tetrabromophenol blue will change from yellow to blue, and the depth of color is positively correlated with the protein concentration. Zhao *et al.* reported,^98^ as shown in Fig. 14(A), the measurement of a fluorescence signal using a self-assembled LIF device. Lasers that expose optical fibers to paper-based chips are emitted by semiconductor laser light sources. Hao Fu *et al.*^22^ developed a new type of controlled fluid valve driven by sheet-molded plastic for fluid operation on μPAD, which is used to control on-board valve operation and telemedicine, quantify colorimetric signal output, display test results, and wirelessly transmit data to smart phones. Fig. 14(B)(a) shows a photograph of the colorimetric reader that accommodates a μPAD for activation of the valves and readout of the colorimetric signal. Fig. 14(B)(b) shows a decomposition diagram of the combined structure of the substrate and operating unit of the colorimetric reader. As shown in Fig. 14(C), based on the principle of self-assembly and self-decomposition of the ionic liquid on the surface of gold electrode, Xun Wu *et al.*^146^ proposed a special self-powered, low-cost green thermoelectric paper chip for fire alarms. This group used the signal thermoelectric unit and thermoelectric module in series of [EMIm][Ac] (p-type) and [EMIm][TFSI] (n-type) thermoelectric units to manufacture the ionic thermoelectric paper chip. When the thermoelectric paper converter is engaged with the temperature gradient, a potential difference between the hot electrode and the cold electrode is generated. The thermoelectric voltage is defined as the potential at the hot electrode minus the potential at the cold electrode. The [EMIm][Ac] and [EMIm][TFSI] thermoelectric converters generate positive and negative voltages, respectively (Table 7).

**Table 7:** Paper chip detection methods

Range of application		Samples	Detection method	Paper used	Production methods	Characteristics	Ref.	
Health detection	Drug screening	Drug screening	Fluorescence detection method	Whatman paper #1	Plasma treatment	The embedded drug gradient generator through paper fluidic network can reduce the reagent consumption, automate the drug gradient generation procedure, and thereby improve the efficiency, while reducing the cost of traditional cell-based bioassays using multi-well plates.	47	47, 48 and 121
		Drug identification	Colorimetric method	Whatman paper #3	Superposition method	A semi-quantitative analysis of artesunate in counterfeit antimalarial drugs can be performed.	120	
	Pathogenic bacteria detection	Cortisol detection	—	—	Surface-enhanced Raman	No additional redox medium is required for electron exchange.	135	14, 66, 127, 135 and 143
		*E. coli*	Electrochemical detection	Mineral paper layer	Process knife cutting method	The accuracy of peak distribution was improved by adding the scaffold step for capillary electrophoresis analysis. It is simple to manufacture, light weight, disposable and low cost.	66	
			Colorimetric method	Whatman paper	—	Simple, fast, specific qualitative and quantitative.	14	
	Disease diagnosis	Carcinoembryonic antigen	Chemiluminescence	Whatman paper #1	Plasma treatment	The amino and aldehyde groups of the antibody covalently conjugate to form a base so that the antibody can be fixed directly on the surface of the paper.	48	48, 51, 52, 60, 64, 65, 83, 98, 100, 105, 121, 133, 136, 141 and 142
			Electrochemical detection	Whatman chromatography filter paper	Melt wax soaking technique	The direct detection of antigens by molecular imprinting on paper-based equipment greatly reduces the cost of clinical detection, and has the advantages of cheap, easy preparation, disposable and reliable analysis.	100	
		Biomarker	Chemiluminescence	Whatman tomographic paper #1	Process knife cutting method	A sugar barrier was established on a paper-based chip microchannel to control the reagent migration rate and reagent transport technology.	105	
		Antigen	Immune detection	Cellulose nitrate	Lateral flow mode paper chips	Using smart phone assisted paper-based microfluidic chip can detect influenza a more conveniently and efficiently	142	
		AFP	Fluorescence detection method	Whatman tomographic paper #1	Wax printing method	The method was used to linearly detect alpha-fetoprotein in the range of 0.001 ng mL^−1^ to 20.0 ng mL^−1^, and the detection limit was 0.4 pg mL^−1^.	98	
Food detection	Detection of pesticides	DDV	Photochemical detection	Whatman tomographic paper #3	Ion imprinting technique	There is no need to activate the paper surface, the detection limit reaches the nanogram level, and the linear response range is the microgram level.	111 and 130	60, 70, 84, 85, 104, 111, 114, 115 and 130
		Pesticide	Colorimetric	Filter paper	Inkjet printing	Organophosphorus hydrolase was used for environmental sensing of pesticides.	70	
			Mass spectrometric detection	—	Wax printing	According to the different reaction efficiency of pesticide molecules, pesticide identification on chip is carried out in chronological order using the reflected light intensity spectrum, and the optimal temperature of enzyme activity is ensured.	85	
	Additive detection	SO_2_	Colorimetric	Whatman paper #1	—	Providing an accurate, low-cost and reliable method for sulfur dioxide detection and has considerable potential for proof-of-concept applications.	121	96, 115 and132
			Colorimetric	Cellulose paper	Melt wax soaking technique	In this method, the effect of structure on the uniformity of detection is studied by means of gray scale analysis.	99	
	Quality inspection	Amylose content in rice	Colorimetric	Whatman filter paper	Plasma treatment	The colorimetric reaction between conventional starch and iodide was used. It is not a substitute for standard methods.	49	47
Environmental	Heavy metal detection	Preconcentration and separation of metal ions	Electrochemical detection	Whatman paper #1	UV lithography; superposition method	The detection limit of Cr^2+^, Cu^2+^, Ni^2+^, and Co^2+^ reached the microgram level, and the detection limit of Fe^3+^ reached the nanogram level.	144	43, 111, 111–113, 123 and 144
		Cd^2+^, Pb^2+^, Cu^2+^ and Hg^2+^	Fluorescence detection	Fiberglass paper	Ion imprinting technique	Cd^2+^ and Pb^2+^ were specifically detected.	112	
	Component detection	Filtered concentrated explosive	Fluorescence detection	Whatman filter paper # 5	Wax printing	The detection range of the eight explosives analyzed was 1.4–5.6 ng, and the paper recovery range was 65–82%.	93	41, 86, 93, 125 and 136
		Wastewater	Photochemical detection	Whatman paper chromatography	Surface-enhanced Raman	Raman spectroscopy is a direct, nondestructive analysis method that requires no additional preparation and only a small amount of sample.	136	
		Chlorine ion in water	Potential detection	Whatman paper #1	UV lithography	Low cost, portable, disposable, no need for complex instruments.	41	
Biochemistry	Biological detection	Separate proteins containing different substances	Electrochemical detection	No. 3, filter paper	Plasma treatment	The simultaneous isolation and enrichment of protein samples containing myoglobin and cytochrome C were realized.	44	18, 42, 44–46, 83, 88, 89, 111, 128, 133, 136 and 137
		Biomacromolecule	Fluorescence detection	Fiberglass paper	Cutting method	The glass fibre-based microfluidic chip has multiple micropores for nucleic acid and protein detection and can simultaneously detect and read targets.	128	
		Nucleic acid testing (NAT)	Fluorescence detection	Fusion 5 filter paper	Plasma treatment	Low cost, rapid and automated extraction of DNA and PCR amplification. DNA was purified from 0.25–1 L human blood to 8.1–21.8 ng DNA.	46	
		Antibody	Mass spectrometric detection	Whatman tomographic paper #1	Wax printing	Satisfactory protein reduction, alkylation and hydrolysis were completed in the sampling facility within 3 h from the sampling stage to the sampling time. Showing high performance of 10–1000 ng mL^−1^.	133	
		Protein	Chromatography	Whatman filter paper	Spray wax printing technology	A new integrated paper-based sampling concept for bottom-up protein analysis by mass spectrometry, which is a full-device chip form that integrates instant immune capture, protein reduction, alkylation, and trypsin digestion.	83	
	Development research	Biological molecules	Electrochemical detection	Whatman tomographic paper #1	Screen printing technology	GN-HPMNS was synthesized and used as an effective immobilized matrix for the preparation of solid-state sensors, and it is more advantageous to detect compounds containing tertiary amino acid and deoxyribonucleic acid.	57	22, 24, 57, 62, 98, 109 and 145
		Production of electrodes	Photochemical detection	Inkjet photo paper	Chemical vapor deposition	All that is needed is a ballpoint pen filled with ink made of conductive material and a digital plotter for printing electrode arrays.	62	

**Fig. 15:**
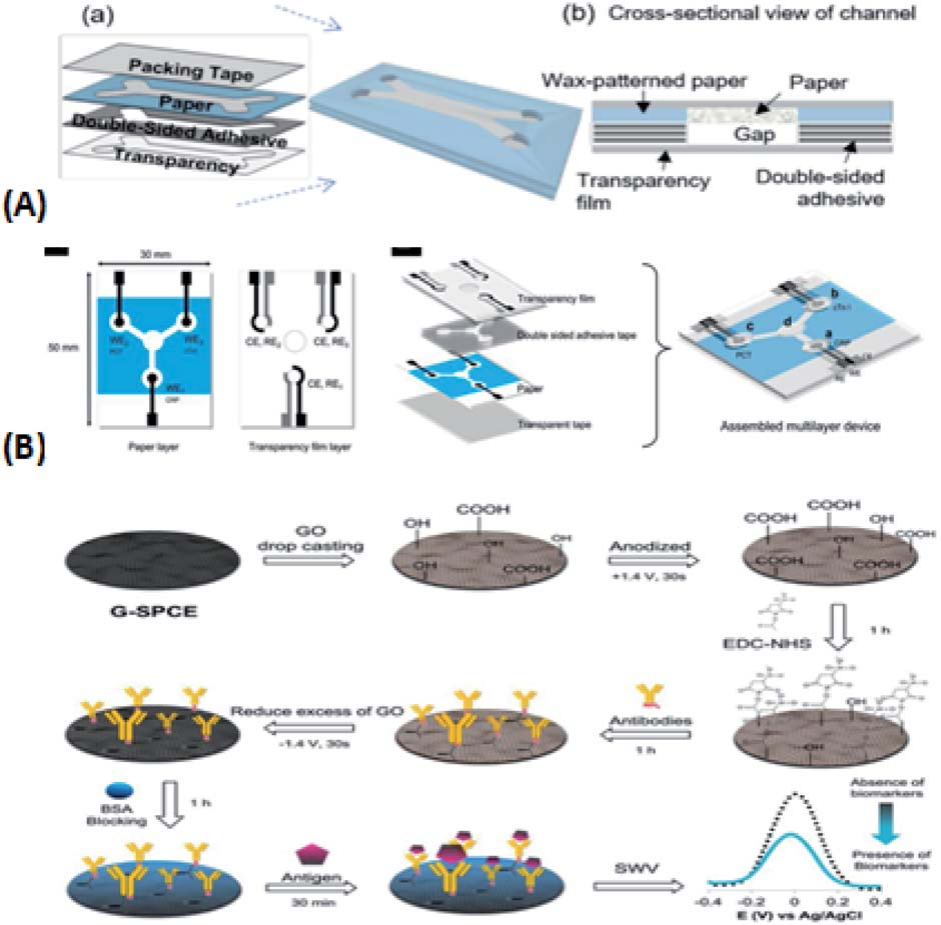
(A) Assembly and flow characteristics of microfluidic paper-based analytical devices (μPADs). (a) Schematic of fast-flow μPAD assembly. (b) Cross-sectional view of channel of fast flow μPADs. (B) Design and fabrication of the multiplexed ePADs and step-by-step preparation of the electrochemical immunosensor and detection of the antigen/biomarker on the sensor.

Charles S. Henry's group summarized some of the tests and applications of paper chip devices in 2020. Here are the latest developments in the group's research.^147^ In a recent paper chip study, Charles S. Henry's group^148^ made the latest findings, where in 2020, they that the magnetophilic capability of a paper-based, fast-flowing microfluidic analysis device (ffPADs) was used to isolate bacteria from complex matrices, eliminating interference from other substances, focusing on target cells, and thus improving detection performance. This technology effectively addresses the shortcomings of traditional microfluidic devices, which require mechanical pumps to drive fluid, improves portability, and makes the transition to a point of care (POC) setting much easier. ffPADs utilize the capillary action in the gap between the stacked layers of paper and transparent sheets to facilitate flow at a higher rate than conventional μPADs. Multilayer ffPADs allow particles and cells to pass through gaps without being captured by the paper layer. The ability of the paper based pumpless magnetophoresis device was also demonstrated by detecting *E. coli* and other bacteria and viruses in urine. Simultaneously, the application prospect of this device in food safety and human health diagnosis in the future was proven. As shown in Fig. 15(A)(a), the unit is assembled by stacking packaging tape, paper, double-sided adhesive, and a transparent material. Fig. 15(A)(b) shows a cross-sectional view of the passage once the device is assembled. In 2021, Charles S. Henry's group reported^149^ an electrochemical paper-based analytical device (ePAD) combined with unlabeled immunoassays, demonstrating an electron probe combined with unlabeled immunoassays. The device is based on an electron probe that passes through a stack of wax paper and a transparent membrane and then uses a laser-cut double-sided adhesive to secure the sample inlet, three separate test zones of the analyte, and a fluid channel that connects the sample inlet to the test zone. When a biomarker is added, antibodies to the biomarker itself are fixed to a carbon electrode modified with graphene oxide, which is printed on the electron probe *via* a template. Finally, the concentration of biomarkers is determined by square wave voltammetry (SWV). The device allows a single device to simultaneously measure C-reactive protein (CRP), troponin I (cTnI), and procalcitonin (PCT), three important biomarkers of cardiovascular disease. Electrochemical changes were measured to detect the presence of these three biomarkers. The current response generated by the combination of the analyte and its fixed antibody was determined. A significant reduction in current response was observed in the presence of cardiac biomarkers, while no change was observed in the absence of biomarkers. As shown in Fig. 15(B), the 4 mm diameter hole made on the transparent film in the image is used to fix the detection antibody and the 5 mm diameter hole is the redox solution inlet, where WE = working electrode; CE = reverse electrode; and RE = reference electrode.

Wendell K.T. Coltro's group^150^ reported significant advances in microfluidic paper chip devices for diagnostic and clinical analysis several years ago. μPads have been developed for many diagnostic applications, including blood typing and detection of molecular biomarkers and microorganisms such as bacteria, viruses and fungi. Earlier, this group^151^ first developed a visual colorimetric paper-based analysis device for detecting procaine in seized samples of the drug cocaine. The principle of this device is to use the diazotization of procaine and the coupling reaction of procaine with acid under alkaline conditions to detect procaine. In the same year,^152^ to improve the quantitative determination of biological enzymes *via* colorimetric determination and for the related analysis of the performance, Wendell K.T. Coltro's group proposed chitosan-modified μPADs for the sensitive colorimetric detection of the glucose levels in tears. They demonstrated that chitosan plays an important role and the introduction of chitosan for the direct electron transfer between the enzymes and active surface provides a suitable microenvironment and forms a better adsorption enzyme solid support. In the presence of potential interference, the performance of the proposed device was evaluated by detecting glucose and uric acid in artificial serum samples to measure the glucose levels in tears, and the feasibility of this method was verified. The improved μPads provide reliability and accuracy for sample analysis. In 2020, Wendell K.T. Coltro's group^153^ proposed a simple new device for the colorimetric measurement of drinking water *via in situ* analysis using a smartphone camera and μPad. The device is fixed to a designed 3D printing stand, and then the cutting printer can quickly and cheaply produce μPAD. Simply, it is a paper chip that uses colorimetry to detect water hardness, phenols and pH values for environmental analysis. Also in 2020, this group^154^ first described using a 3D pen with no other instrument or equipment for making a paper-based analysis device for pollution and water quality monitoring, clinical application and quantitative determination of environmental samples. The production steps only need to use 3D pen painting and directly use a portable flashlight for UV curing in two simple steps. This device has one of the most obvious advantage that is compatible with organic solvents, and when exposed to different chemical samples such as surfactants, alkali solution and organic solvents (except ethanol), it is chemical resistance.

## 11 Summary

As a hotspot in the field of analytical chemistry detection, microfluidic paper chip technology has been researched and applied in various fields, ranging from the original test paper analysis to the two-dimensional microflow system and then the three-dimensional flow system. Testing and analysis on only a few square centimeters of paper-based materials can greatly save testing time and reduce the consumption of reagents and samples, and simultaneously protect the environment, and relatively accurate chemical means can be used for substance detection. As an increasing number of researchers study paper chips, the production process and detection technology of paper chips become simpler and more accurate, and their applications become more diverse. However, although extensive use of paper chips in various fields is desirable, they still have many defects to be solved. For example, because the printing materials on the paper-based materials are very fragile, they are easily destroyed during folding, bending and tearing. Although appropriate paper-based materials can be selected to alleviate this problem, the reproducibility and stability of paper chips are still not ideal. The colorimetric method is the most commonly used detection method. However, there is no good preservation condition and relatively stable transportation mode in long-distance transportation, its sensitivity will be affected and the accuracy will be reduced. The bigger challenge is that paper chips can reduce costs and eliminate cross-contamination, though in biochemistry it is not possible to test the samples directly. Instead, a large amount of time is needed to process the samples manually and then complete the test by using paper chips. With the continuous research of analysis technology and the continuous expansion of paper chip technology, the structure design of suitable paper chips will also be constantly developed, and their production process will have a broad prospect. With the modification of advanced materials and the selection of appropriate sensitive detection methods, paper chips will play a more important role in the fields of health detection, food detection, environmental detection and biochemistry.

## Conflicts of interest

There are no conflicts to declare.

## Supplementary Material

## References

[cit1] Terry S. C., Jerman J. H., Angell J. B. (1979). A gas chromatographic air analyzer fabricated on a silicon wafer. IEEE Trans. Electron Devices.

[cit2] Berthier E., Young E. W. K., Beebe D. (2012). Engineers are from PDMS-land, Biologists are from Polystyrenia. Lab Chip.

[cit3] Chu C., Jiang B., Zhu L. (2015). *et al.*, A process analysis for microchannel deformation and bonding strength by in-mold bonding of microfluidic chips. J. Polym. Eng..

[cit4] Pan Y.-J., Yang R.-J. (2006). A glass microfluidic chip adhesive bonding method at room temperature. J. Micromech. Microeng..

[cit5] Riegger L., Strohmeier O., Faltin B. (2010). *et al.*, Adhesive bonding of microfluidic chips: influence of process parameters. J. Micromech. Microeng..

[cit6] Zeng F., Mou T., Zhang C. (2018). *et al.*, Paper-based SERS analysis with smartphones as Raman spectral analyzers. Analyst.

[cit7] Dal Dosso F., Bondarenko Y., Kokalj T. (2019). *et al.*, SIMPLE analytical model for smart microfluidic chip design. Sens. Actuators, A.

[cit8] Tabeling P. (2014). Recent progress in the physics of microfluidics and related biotechnological applications. Curr. Opin. Biotechnol..

[cit9] Baharloo M., Aligholipour R., Abdollahi M. (2020). *et al.*, A power efficient multiple network-on-chip architecture. Computers and Electrical Engineering.

[cit10] Zheng X., Chen L., Wang B. (2020). *et al.*, Novel fairness-aware co-scheduling for shared cache contention game on chip multiprocessors. Inf. Sci..

[cit11] Manz A., Graber N., Widmer H. M. (1990). Miniaturized Total Chemical Analysis Systems: a Novel Concept for Chemical Sensing. Sens. Actuators, B.

[cit12] Bhakta S. A., Borba R., Taba Jr M. (2014). *et al.*, Determination of nitrite in saliva using microfluidic paper-based analytical devices. Anal. Chim. Acta.

[cit13] Liu H., Li X., Crooks R. M. (2013). Paper-based SlipPAD for high-throughput chemical sensing. Anal. Chem..

[cit14] Dungchai W., Sameenoi Y., Chailapakul O. (2013). *et al.*, Determination of aerosol oxidative activity using silver nanoparticle aggregation on paper-based analytical devices. Analyst.

[cit15] Amziah Md Yunus N., Ismail N. F., Abdul Halin I. (2018). *et al.*, Microdroplet electrowetting actuation on flexible paper-based lab on a chip. Results Phys..

[cit16] Daniel M. C., Astruc D. (2004). Gold nanoparticles: assembly, supramolecular chemistry, quantum-size-related properties, and applications toward biology, catalysis, and nanotechnology. Chem. Rev..

[cit17] Lin M., Zhao Y., Wang S. Q. (2012). *et al.*, Recent advances in synthesis and surface modification of lanthanide-doped upconversion nanoparticles for biomedical applications. Biotechnol. Adv..

[cit18] Fu Y., Zhou X., Xing D. (2018). Integrated paper-based detection chip with nucleic acid extraction and amplification for automatic and sensitive pathogen detection. Sens. Actuators, B.

[cit19] Cate D. M., Dungchai W., Cunningham J. C. (2013). *et al.*, Simple, distance-based measurement for paper analytical devices. Lab Chip.

[cit20] Erickson D., O'Dell D., Jiang L. (2014). *et al.*, Smartphone technology can be transformative to the deployment of lab-on-chip diagnostics. Lab Chip.

[cit21] Martinez A. W., Phillips D. S. T., Butte D. M. J. (2007). *et al.*, Patterned paper as a platform for inexpensive, low-volume, portable bioassays. Angew. Chem., Int. Ed..

[cit22] Fu H., Song P., Wu Q. (2019). *et al.*, A paper-based microfluidic platform with shape-memory-polymer-actuated fluid valves for automated multi-step immunoassays. Microsyst. Nanoeng..

[cit23] Biro D. a., Pleizier G., Yves D. (1993). Application of the Microbond Technique IV. Improved. Appl. Polym. Sci..

[cit24] Monsur Ali M., Aguirre S. D., Xu Y. (2009). *et al.*, Detection of DNA using bioactive paper strips. Chem. Commun..

[cit25] Martinez A. W., Phillips S. T., Whitesides G. M. (2010). Diagnostics for the Developing World: Microfluidic Paper-Based Analytical Devices. Anal. Chem..

[cit26] Roger E. L., Brennan J. D. (2010). Bioactive Paper Dipstick Sensors for Acetylcholinesterase Inhibitors Based on Sol-Gel/Enzyme/Gold Nanoparticle Composites. R. Soc. Chem..

[cit27] Schilling K. M. (2012). *et al.*, Fully enclosed microfluidic paper-based analytical devices. Anal. Chem..

[cit28] Schilling K. M., Lepore A. L., Kurian J. A. (2015). *et al.*, Detection of water contamination from hydraulic fracturing wastewater: a muPAD for bromide analysis in natural waters. Analyst.

[cit29] Yang S.-T., Cao L., Luo P. G. (2009). *et al.*, Carbon dots for optical imaging in vivo. J. Am. Chem. Soc..

[cit30] Rolland J. P., Mourey D. A. (2013). Paper as a novel material platform for devices. MRS Bull..

[cit31] Whitesides G. M. (2013). Cool, or simple and cheap? Why not both?. Lab Chip.

[cit32] Toley B. J., McKenzie B., Liang T. (2013). *et al.*, Tunable-delay shunts for paper microfluidic devices. Anal. Chem..

[cit33] Whitesides G. M. (2013). Viewpoint on "Dissolvable fluidic time delays for programming multi-step assays in instrument-free paper diagnostics". Lab Chip.

[cit34] Jang I., Song S. (2015). Facile and precise flow control for a paper-based microfluidic device through varying paper permeability. Lab Chip.

[cit35] Camplisson C. K., Schilling K. M., Pedrotti W. L. (2015). *et al.*, Two-ply channels for faster wicking in paper-based microfluidic devices. Lab Chip.

[cit36] Klasner S. A., Price A. K., Hoeman K. W. (2010). *et al.*, Paper-based microfluidic devices for analysis of clinically relevant analytes present in urine and saliva. Anal. Bioanal. Chem..

[cit37] Martinez A. W., Phillips S. T., Nie Z. (2010). *et al.*, Programmable diagnostic devices made from paper and tape. Lab Chip.

[cit38] Carrilho E., Phillips S. T., Vella S. J. (2009). *et al.*, Paper Microzone Plates. Anal. Chem..

[cit39] Martinez A. W., Phillips S. T., Wiley B. J. (2008). FLASH A Rapid Method for Prototyping Paper-Based Microfluidic Devices. Lab Chip.

[cit40] Martinez A. W., Phillips S. T., Carrilho E. (2008). *et al.*, Simple telemedicine for develop-ing regions: camera phones and paper-based microfluidic devices for real-time, off-site diagnosis. Anal. Chem..

[cit41] Liu H., Crooks R. M. (2011). Three-dimensional paper microfluidic devices assembled using the principles of origami. J. Am. Chem. Soc..

[cit42] Lou B., Chen C., Zhou Z. (2013). *et al.*, A novel electrochemical sensing platform for anions based on conducting polymer film modified electrodes integrated on paper-based chips. Talanta.

[cit43] Sun H., Yuan J., Dong H. (2018). *et al.*, Multiplex quantification
of metals in airborne particulate matter via smartphone and paper-based microfluidics. Anal. Chim. Acta.

[cit44] Li X., Tian J., Shen W. (2010). Progress in patterned paper sizing for fabrication of paper-based microfluidic sensors. Cellulose.

[cit45] Yu S., Yan C., Hu X. (2019). *et al.*, Isoelectric focusing on microfluidic paper-based chips. Anal. Bioanal. Chem..

[cit46] Gan W., Zhuang B., Zhang P. (2014). *et al.*, A filter paper-based microdevice for low-cost, rapid, and automated DNA extraction and amplification from diverse sample types. Lab Chip.

[cit47] Hong B., Xue P., Wu Y. (2016). *et al.*, A concentration gradient generator on a paper-based microfluidic chip coupled with cell culture microarray for high-throughput drug screening. Biomed. Microdevices.

[cit48] Zhao M., Li H., Liu W. (2016). *et al.*, Plasma treatment of paper for protein immobilization on paper-based chemiluminescence immunodevice. Biosens. Bioelectron..

[cit49] Hu X., Lu L., Fang C. (2015). *et al.*, Determination of Apparent Amylose Content in Rice by Using Paper-Based Microfluidic Chips. J. Agric. Food Chem..

[cit50] Chen Y., Chu W., Liu W. (2018). *et al.*, Distance-based carcinoembryonic antigen assay on microfluidic paper immunodevice. Sens. Actuators, B.

[cit51] Chen Y., Chu W., Liu W. (2018). *et al.*, Paper-based chemiluminescence immunodevice for the carcinoembryonic antigen by employing multi-enzyme carbon nanosphere signal enhancement. Mikrochim. Acta.

[cit52] Abe K., Kotera K., Suzuki K. (2010). *et al.*, Inkjet-printed paperfluidic immuno-chemical sensing device. Anal. Bioanal. Chem..

[cit53] Abe K., Daniel Citterio K. S. (2008). Inkjet-Printed Microfluidic Multianalyte Chemical. Anal. Chem..

[cit54] Dungchai W., Chailapakul O., Henry C. S. (2011). A low-cost, simple, and rapid fabrication method for paper-based microfluidics using wax screen-printing. Analyst.

[cit55] Nie Z., Nijhuis C. A., Gong J. (2010). Electrochemical Sensing in Paper-Based Microfluidic Devices. Lab Chip.

[cit56] Yafia M., Shukla S., Najjaran H. (2015). Fabrication of digital microfluidic devices on flexible paper-based and rigid substrates via screen printing. J. Micromech. Microeng..

[cit57] Xu Y., Lv Z., Xia Y. (2013). *et al.*, Highly porous magnetite/graphene nanocomposites for a solid-state electrochemiluminescence sensor on paper-based chips. Anal. Bioanal. Chem..

[cit58] Liu H., Zhou X., Shen Q. (2018). *et al.*, Paper-based electrochemiluminescence sensor for highly sensitive detection of amyloid-beta oligomerization: Toward potential diagnosis of Alzheimer's disease. Theranostics.

[cit59] Ali R., Rodrigues A. D., Holzmann D. (2020). Thermosonic fine-pitch flipchip bonding of silicon chips on screen printed paper and PET substrates. Microelectron. Eng..

[cit60] Wu F., Wang M. (2018). A Portable Smartphone-Based Sensing System Using a 3D-Printed Chip for On-Site Biochemical Assays. Sensors.

[cit61] Bruzewicz D. A., Reches M., Whitesides G. M. (2008). Low-cost printing of poly(dimethylsiloxane) barriers to define microchannels in paper. Anal. Chem..

[cit62] Soum V., Kim Y., Park S. (2019). *et al.*, Affordable Fabrication of Conductive Electrodes and Dielectric Films for a Paper-based Digital Microfluidic Chip. Micromachines.

[cit63] Ye X., Xu J., Lu L. (2018). *et al.*, Equipment-free nucleic acid extraction and amplification on a simple paper disc for point-of-care diagnosis of rotavirus A. Anal. Chim. Acta.

[cit64] Chen Y., Wang J., Liu Z. (2018). *et al.*, A simple and versatile paper-based electrochemilumine-scence biosensing platform for hepatitis B virus surface antigen detection. Biochem. Eng. J..

[cit65] Erin M. F., Mascarenas M. R., Gabriel P. L. (2009). *et al.*, Multiplex lateral-flow test strips fabricated by two-dimensional shaping. ACS Appl. Mater. Interfaces.

[cit66] Lee J. W., Lee D., Kim Y. T. (2017). *et al.*, Low-cost and facile fabrication of a paper-based capillary electrophoresis microdevice for pathogen detection. Biosens. Bioelectron..

[cit67] Fu E., Lutz B., Kauffman P. (2010). *et al.*, Controlled reagent transport in disposable 2D paper networks. Lab Chip.

[cit68] Mao X., Huang T. J. (2012). Microfluidic diagnostics for the developing world. Lab Chip.

[cit69] Chitnis G., Ding Z., Chang C.-L. (2011). *et al.*, Laser-treated hydrophobic paper: an inexpensive microfluidic platform. Lab Chip.

[cit70] Jafry A. T., Lee H., Pradhipta Tenggara A. (2019). *et al.*, Double-sided electro- hydrodynamic jet printing of two-dimensional electrode array in paper-based digital microfluidics. Sens. Actuators, B.

[cit71] Jemmeli D., Marcoccio E., Moscone D. (2020). *et al.*, Highly sensitive paper-based electrochemical sensor for reagent free detection of bisphenol A. Talanta.

[cit72] Carter J. C., Alvis R. M., Brown S. B. (2005). *et al.*, Fabricating Optical Fiber Imaging. Biosens. Bioelectron..

[cit73] Hayes D. J. , Royall Cox W. and Grove M. E., Low-Cost Display Assembly and Interconnect Using Ink-Jet Printing Technology. MicroFab Technologies, Inc, 2001

[cit74] Hue P. Le (1998). Progress and Trends in Ink-jet. J. Imaging Sci. Technol..

[cit75] Nie Z., Deiss F., Liu X. (2010). *et al.*, Integration of paper-based microfluidic devices with commercial electrochemical readers. Lab Chip.

[cit76] Feng Q., Chen H., Xu J. (2015). Disposable paper-based bipolar electrode array for multiplexed electrochemiluminescence detection of pathogenic DNAs. Sci. China: Chem..

[cit77] Pardee K., Green A. A., Ferrante T. (2014). *et al.*, Paper-based Synthetic Gene Networks. Cell.

[cit78] Lu Y., Shi W., Jiang L. (2009). *et al.*, Rapid prototyping of paper-based microfluidics with wax for low-cost, portable bioassay. Electrophoresis.

[cit79] Lu Y., Lin B., Qin J. (2011). Patterned paper as a low-cost, flexible substrate for rapid prototyping of PDMS microdevices via "liquid molding". Anal. Chem..

[cit80] Jokerst J. C., Adkins J. A., Bisha B. (2012). *et al.*, Development of a paper-based analytical device for colorimetric detection of select foodborne pathogens. Anal. Chem..

[cit81] Zhang Y., Zuo P., Ye B. C. (2015). A low-cost and simple paper-based microfluidic device for simultaneous multiplex determination of different types of chemical contaminants in food. Biosens. Bioelectron..

[cit82] Matsuura H., Ujiie K., My Duyen T. T. (2019). *et al.*, Development of a Paper-Based Luminescence Bioassay for Therapeutic Monitoring of Aminoglycosides: a Proof-of-Concept Study. Appl. Biochem. Biotechnol..

[cit83] Skjaervo O., Halvorsen T. G., Reubsaet L. (2019). All-in-one paper-based sampling chip for targeted protein analysis. Anal. Chim. Acta.

[cit84] Jin L., Zhenxia H., Zheng Q. (2020). *et al.*, A facile microfluidic paper-based analytical device for acetylcholinesterase inhibition assay utilizing organic solvent extraction
in rapid detection of pesticide residues in food. Anal. Chim. Acta.

[cit85] Yang N., Shaheen N., Xie L. (2019). *et al.*, Pesticide Residues Identification by Optical Spectrum in the Time-Sequence of Enzyme Inhibitors Performed on Microfluidic Paper-Based Analytical Devices (microPADs). Molecules.

[cit86] Liu C.-C., Wang Y.-N., Fu L.-M. (2018). *et al.*, Microfluidic paper-based chip platform for formaldehyde concentration detection. Chem. Eng. J..

[cit87] Liu C.-C., Wang Y.-N., Fu L.-M. (2018). *et al.*, Microfluidic paper-based chip platform for benzoic acid detection in food. Food Chem..

[cit88] Fu L.-M., Tseng C.-C., Ju W.-J. (2018). *et al.*, Rapid Paper-Based System for Human Serum Creatinine Detection. Inventions.

[cit89] Wang X., Sun J., Tong J. (2018). *et al.*, Paper-Based Sensor Chip for Heavy Metal Ion Detection by SWSV. Micromachines.

[cit90] Yang R.-J., Tseng C.-C., Ju W.-J. (2018). *et al.*, Integrated microfluidic paper-based system for determination of whole blood albumin. Sens. Actuators, B.

[cit91] Zhang L., Sun L., Hou M. (2018). *et al.*, A paper-based photothermal array using Parafilm to analyze hyperthermia response of tumour cells under local gradient temperature. Biomed. Microdevices.

[cit92] Yang R.-J., Tseng C.-C., Ju W.-J. (2018). *et al.*, A rapid paper-based detection system for determination of human serum albumin concentration. Chem. Eng. J..

[cit93] Ueland M., Blanes L., Taudte R. V. (2016). *et al.*, Capillary-driven microfluidic paper-based analytical devices for lab on a chip screening of explosive residues in soil. J. Chromatogr. A.

[cit94] Liu P., Li B., Fu L. (2020). *et al.*, Hybrid Three Dimensionally Printed Paper-Based Microfluidic Platform for Investigating a Cell's Apoptosis and Intracellular Cross-Talk. ACS Sens..

[cit95] Yehia A. M., Farag M. A., Tantawy M. A. (2020). A novel trimodal system on a paper-based microfluidic device for on-site detection of the date rape drug "ketamine. Anal. Chim. Acta.

[cit96] Cai X., Zhang H., Yu X. (2020). *et al.*, A microfluidic paper-based laser-induced fluorescence sensor based on duplex-specific nuclease amplification for selective and sensitive detection of miRNAs in cancer cells. Talanta.

[cit97] Huang G.-W., Feng Q.-P., Xiao H.-M. (2016). *et al.*, Rapid Laser Printing of Paper-Based Multilayer Circuits. ACS Nano.

[cit98] Zhao M., Li H., Liu W. (2017). *et al.*, Paper-based laser induced fluorescence immunodevice combining with CdTe embedded silica nanoparticles signal enhancement strategy. Sens. Actuators, B.

[cit99] Fu L.-M., Liu C.-C., Yang C.-E. (2019). *et al.*, A PET/paper chip platform for high resolution sulphur dioxide detection in foods. Food Chem..

[cit100] Qi Ji, Li B., Zhou N. (2019). *et al.*, The strategy of antibody-free biomarker analysis by in situ synthesized molecularly imprinted polymers on movable valve paper-based device. Biosens. Bioelectron..

[cit101] Zhao W., Ali M. M., Aguirre S. D. (2008). *et al.*, Flexographically Printed Fluidic Structures in. Anal. Chem..

[cit102] Demirel G., Babur E. (2014). Vapor-phase deposition of polymers as a simple and versatile technique to generate paper-based microfluidic platforms for bioassay applications. Analyst.

[cit103] Haller P. D., Flowers C. A., Gupta M. (2011). Three-dimensional patterning of porous materials using vapor phase polymerization. Soft Matter.

[cit104] Yang N., Zhou X., Yu D. (2020). *et al.*, Pesticide residues identification by impedance time-sequence. J. Food Process Eng..

[cit105] Chu W., Chen Y., Liu W. (2017). *et al.*, Paper-based chemiluminescence immunodevice with temporal controls of reagent transport technique. Sens. Actuators, B.

[cit106] Lewis G. G., DiTucci M. J., Baker M. S. (2012). *et al.*, High throughput method for prototyping three-dimensional, paper-based microfluidic devices. Lab Chip.

[cit107] Martinez A. W., Phillips S. T., Whitesides G. M. (2008). Three-dimensional microfluidic devices fabricated. Proc. Natl. Acad. Sci. U. S. A..

[cit108] Martinez A. W., Phillips S. T., Nie Z. (2010). Programmable Diagnostic Devices Made from Paper and Tape. Lab Chip.

[cit109] Chen P., Chen C., Liu Y. (2019). *et al.*, Fully integrated nucleic acid p,retreatment, amplification, and detection on a paper chip for identifying EGFR mutations in lung cancer cells. Sens. Actuators, B.

[cit110] Xiao L., Liu X., Zhong R. (2013). *et al.*, A rapid, straightforward, and print house compatible mass fabrication method for integrating 3D paper-based microfluidics. Electrophoresis.

[cit111] Liu W., Guo Y., Luo J. (2015). *et al.*, A molecularly imprinted polymer based a lab-on-paper chemiluminescence device for the detection of dichlorvos. Spectrochim. Acta, Part A.

[cit112] Qi J., Li B., Wang X. (2017). *et al.*, Three-dimensional paper-based microfluidic chip device for multiplexed fluorescence detection of Cu2+ and Hg2+ ions based on ion imprinting technology. Sens. Actuators, B.

[cit113] Zhou J., Li B., Qi A. (2020). *et al.*, ZnSe quantum dot based ion imprinting technology for fluorescence detecting cadmium and lead ions on a three-dimensional rotary paper-based microfluidic chip. Sens. Actuators, B.

[cit114] Zhao P., Liu H., Zhang L. (2020). *et al.*, Paper-Based SERS Sensing Platform Based on 3D Silver Dendrites. ACS Appl. Mater. Interfaces.

[cit115] Guoying H., Zhang Z., Ma X. (2020). *et al.*, A versatile microfluidic paper chip platform based on MIPs for rapid. Microchem. J..

[cit116] Vella S. J., Beattie P., Cademartiri R. (2012). *et al.*, Measuring markers of liver function using a micropatterned paper device designed for blood from a fingerstick. Anal. Chem..

[cit117] Martinez A. W., Phillips S. T., Carrilho E. (2008). *et al.*, Simple Telemedicine for Developing Regions. Anal. Chem..

[cit118] Han K. N., Choi J. S., Kwon J. (2017). Gold nanozyme-based paper chip for colorimetric detection of mercury ions. Sci. Rep..

[cit119] Yang N., Chen C., Wang P. (2019). *et al.*, Structure optimization method of microfluidic paper chip based on image grey-level statistics for chromogenic reaction. Chem. Eng. Process..

[cit120] Koesdjojo M. T., Wu Y., Boonloed A. (2014). *et al.*, Low-cost, high-speed identification of counterfeit antimalarial drugs on paper. Talanta.

[cit121] Liu C.-C., Wang Y.-N., Fu L.-M. (2017). *et al.*, Rapid integrated microfluidic paper-based system for sulfur dioxide detection. Chem. Eng. J..

[cit122] Suaifan G., Alhogail S., Zourob M. (2017). Paper-based magnetic nanoparticle-peptide probe for rapid and quantitative colorimetric detection of Escherichia coli O157:H7. Biosens. Bioelectron..

[cit123] Wu C., Gao G., Zhai K. (2020). *et al.*, A visual Hg2+ detection strategy based on distance as readout by Gquadruplex. Food Chem..

[cit124] Zhou C., You T., Jang H. (2020). *et al.*, Aptamer-Conjugated Polydiacetylene Colorimetric Paper Chip for the Detection of Bacillus thuringiensis Spores. Sensors.

[cit125] Gong W., Zhang L., Yu Y. (2020). *et al.*, Modification of Cu3(BTC)2 with Cobalt Ion for Adsorption. Appl. Organomet. Chem..

[cit126] Rong X., Ye-Xin Y., Min L. (2020). *et al.*, Paper-based Microfluidic Chip for Detection of 3-Nitrogen in Water Based on Chromogenic Method. Chin. J. Anal. Chem..

[cit127] Nie Z., Nijhuis C. A., Gong J. (2010). *et al.*, Electrochemical sensing in paper-based microfluidic devices. Lab Chip.

[cit128] Vandaveer IV W. R., Pasas-Farmer S. A., Fischer D. J. (2004). *et al.*, Recent developments in electrochemical detection for microchip capillary electrophoresis. Electrophoresis.

[cit129] Li H., Fang X., Cao H. (2016). *et al.*, Paper-based fluorescence resonance energy transfer assay for directly detecting nucleic acids and proteins. Biosens. Bioelectron..

[cit130] Liu W., Kou J., Xing H. (2014). *et al.*, Paper-based chromatographic chemiluminescence chip for the detection of dichlorvos in vegetables. Biosens. Bioelectron..

[cit131] Sun X., Li B., Tian C. (2018). *et al.*, Rotational paper-based electrochemiluminesc-ence immunodevices for sensitive and multiplexed detection of cancer biomarkers. Anal. Chim. Acta.

[cit132] Adressable TiO, nanotube functionalized paper-based Cyto photoelectric sensor for efficient evaluation of cancer cell surface protein expression.

[cit133] Jang I., Ko H., You G. (2017). *et al.*, Application of paper EWOD (electrowetting-on-dielectrics) chip: Protein tryptic digestion and its detection using MALDI-TOF mass spectrometry. BioChip J..

[cit134] Dou B., Luo Y., Chen X. (2015). *et al.*, Direct measure-ment of beta-agonists in swine hair extract in multiplexed mode by surface-enhanced Raman spectroscopy and microfluidic paper. Electrophoresis.

[cit135] Khan M. S., Misra S. K., Wang Z. (2017). *et al.*, Paper-Based Analytical Biosensor Chip Designed from Graphene-Nanoplatelet-Amphiphilic-diblock-co-Polymer Composite for Cortisol Detection in Human Saliva. Anal. Chem..

[cit136] Lee J.-C., Kim W., Choi S. (2017). Fabrication of a SERS-encoded microfluidic paper-based analytical chip for the point-of-assay of wastewater. Int. J. Precis..

[cit137] Hu S.-W., Qiao S., Pan J.-B. (2018). *et al.*, A paper-based SERS test strip for quantitative detection of Mucin-1 in whole blood. Talanta.

[cit138] Zhang W., Li B., Chen L. (2014). *et al.*, Brush- ing, a simple way to fabricate SERS active paper substrates. Anal. Methods.

[cit139] Wang L., Chen W., Xu D. (2009). *et al.*, Simple, rapid, sensitive, and versatile SWNT-paper sensor for environmental toxin detection competitive with ELISA. Nano Lett..

[cit140] Wu D., Zhang J., Xu F. (2017). *et al.*, A paper-based microfluidic Dot-ELISA system with smartphone for the detection of influenza A. Microfluid. Nanofluid..

[cit141] Li L., Zheng X., Huang Y. (2018). *et al.*, Addressable TiO2 Nanotubes Functionalized paper-based Cyto-Sensor with Photocontrollable Switch for Highly-Efficient Evaluating Surface Protein Expressions of Cancer Cells. Anal. Chem..

[cit142] Ulep T.-H., Ryan Z., Gonzales A. (2020). *et al.*, Smartphone based on-chip fluorescence imaging and capillary flow velocity. Biosens. Bioelectron..

[cit143] Khan M. S., Misra S. K., Dighe K. (2018). *et al.*, Electrically-receptive and thermally-responsive paper-based sensor chip for rapid detection of bacterial cells. Biosens. Bioelectron..

[cit144] Tang R., Yang H., Gong Y. (2017). *et al.*, A fully disposable and integrated paper-based device for nucleic acid extraction, amplification and detection. Lab Chip.

[cit145] Ouyang L., Liu Q., Liang H. (2017). Combining field-amplified sample stacking with moving reaction boundary electrophoresis on a paper chip for the preconcentration and sparation of metal ions. J. Sep. Sci..

[cit146] Wu X., Gao N., Zheng X. (2020). *et al.*, Self-Powered and Green Ionic-Type Thermoelectric Paper Chips for Early Fire Alarming. ACS Appl. Mater. Interfaces.

[cit147] Ozer T., McMahon C., Henry C. S. (2020). Advances in Paper-Based Analytical Devices. Annu. Rev. Anal. Chem..

[cit148] Call Z. D., Carrell C. S., Jang l. (2020). *et al.*, Paper-based pump-free magnetophoresis. Anal. Methods.

[cit149] Boonkaew S., Jang I., Noviana E. (2021). *et al.*, Electrochemical paper-based analytical device for multiplexed,point-of-care detection of cardiovascular disease biomarkers. Sens. Actuators, B.

[cit150] Coltro T., Karlos W., Cheng C.-M. (2014). *et al.*, Recent advances in low-cost microfluidic platforms for diagnostic applications. Electrophoresis.

[cit151] Guedes Silva T., Wendell K., Coltro T., Reis de Araujo W. (2016). *et al.*, Simple and Sensitive Paper-Based Device Coupling Electrochemical Sample Pretreatment and Colorimetric Detection. Anal. Chem..

[cit152] Wendell K. T. C., Garcia E. F. M., Garcia P. T. (2016). *et al.*, Highly sensitive colorimetric detection of glucose and uric acid in biological fluids using chitosan-modified paper microfluidic devices. Analyst.

[cit153] Coltro W. K. T., Janegitz B. C., Pedro da Silva V. A. O. (2020). *et al.*, Microfluidic paper-based device integrated with smartphone for point-of-use colorimetric monitoring of water quality index. Measurement.

[cit154] Sousa L. R., Duarte L. C., Coltro W. K. T. (2020). Instrument-free fabrication of microfluidic paper-based analytical devices through 3D pen drawing. Sens. Actuators, B.

